# Supplementary material for: Identification and characterization of a novel zebrafish (Danio rerio) pentraxin–carbonic anhydrase
Source: PeerJ. 2017 Dec 7;5:e4128. doi: 10.7717/peerj.4128 (PMC5723433; doi:10.7717/peerj.4128)
Supplement: Supplemental Information 7 — Four of the sequences were edited as specified in Table 4. [file peerj-05-4128-s007.pdf]

```
                             *        20         *        40         *        60         *        80
ref|XP_012726090.1| : ---MEILSAFVSAALVCLVN--GGAHTDIHWTYQ-EGALLDQKHWANKYPACGGKKQSPIDIQRSKVKYDEGMLQLELSGY : 74
ref|XP_007570993.1| : ---MEILSAFVSAALVCLVN--AGAHTDIHWTYK-EGALLDQTHWATKYPACGGKKQSPIDIQRRNVRFNQNMLQLELSGY : 74
ref|XP_008411549.1| : ---MEILSAFISAALVCLVN--AGAHSDIHWTYK-EGALLDQMHWATKYPACGGKKQSPIDIQRRNVRFNQNMLQLELTGY : 74
ref|XP_004070439.1| : ---MEVLSAILSALLLRFVA----ADNRIHWTYT-EGALLDQQHWATEYPACAGKKQSPIDIQRRNVKHNPNMLQLELSNY : 72
emb|CAF93821.1|     : ---MELILVFVS-ALVCVV-NAGVHLDGIHWTYK-EGALLDQMHWPTKYPACGGKKQSPIDIQQRNVRFNPDMLQLELSGY : 74
ref|XP_011620264.1| : ---MEQICVFASALFFCVV-SAGVHLDGIHWTYT-EGALLDQMHWPTKYPACGGKQQSPIDIQRRNVRFNPDMLQLKLSGY : 75
ref|XP_013875818.1| : --METLLSAFVSTVFICVV-SAGDPHEDIHWTYK-EGALLDQMHWATKYPECGGRKQSPIDIQRRNVRYNSDMLQLELSGY : 76
ref|XP_005478239.2| : ---MEIFCVFFSAIFAYVV-GDAIPHHGIHWTYK-EGALLDQMHWATQYPACGGKKQSPIDIQRRNVRHNPDMLQLELSGY : 75
ref|XP_006785611.1| : ---MEIFCVFFSALFAYVV-GDAIPHHGIHWTYK-EGALLDQMHWGTQYPACGGKKQSPIDIQRRSVRHNPDLLQLELSGY : 75
ref|XP_005914985.1| : ---MEIFCVFFSALFAYVV-GDAIPHHGIHWTYK-EGALLDQMHWATQYPACGGKKQSPIDIQRRNVRHNPDLLQLEFSGY : 75
ref|XP_004554282.1| : ---MELFCVFFSALFAYVV-GDAIPHHGIHWTYK-EGALLDQMHWATQYPACGGKKQSPIDIQRRNVRHNPDLLQLEFSGY : 75
ref|XP_005733115.1| : ---MELFCVFFSALFAYVV-GDAIPHHGIHWTYK-EGALLDQMHWATQYPACGGKKQSPIDIQRRNVRHNPDLLQLEFSGY : 75
ref|XP_010739228.1| : ----MELSVFVSAFLLCVV-NAGIPHDGIHWTYA-EGALLDQMHWPTKYPSCGGKKQSPIDIQRRRVRHNPDMLQLELNGY : 74
ref|XP_008317045.1| : ---MKLLSLFTTAVWICVA-TAGIRHDDIHWTYT-EGALLDQMHWPTKYPACGGKKQSPIDIQRRKVRHNPDILQLELAGY : 75
ref|XP_010793030.1| : ---MEFLSVFVSSVLICVISAAGIAHDGIHWTYT-EGALLDQMHWPTKYPSCGGKKQSPIDIQRRSVRHNPDMLQLELNGY : 76
ref|XP_008294025.1| : ---MDTLSVFVSAVLVSVV-GAGIPHDGIHWTYT-EGALLDQMHWPTKYPACGGKKQSPIDIQRRSVRHNPDILQLELSGY : 75
ref|XP_002666525.1| : ---MEQLTLVLL--FVTSLNFASAGVDGDYWTYS--GELLDQKHWAEKYHDCGGQQQSPIDIQRRKVRYSPRMQQLELTGY : 73
ref|XP_009295179.1| : ---MEQLTLVLL--FVTSLNFASAGVDGDYWTYS--GELLDQKHWAEKYHDCGGQQQSPIDIQRRKVRYSPRMQQLELTGY : 73
ref|XP_007246352.1| : ---MK---LLA---LLIHVVWVGFSWAGVHWTYQ-ECDLLDQMHWPEKYPSCGGRKQSPIDIQRRSVRFNPHLLQLELTGY : 70
gb|KPP63671.1|      : ------------------------------------------MHWVEKYPACGGRLQSPIDIQRRSVRYNYSLPLLELTGY : 39
ref|XP_012681508.1| : ----------------------------------------MDQMHWAEEYPACGGHKQSPIDIQRRNVRHNPRMPELELTGY : 42
ref|XP_010902628.1| : ---MECFSLFI---HFLLVNVASAGVDGIHWTYS-EGALLDQKHWAKEFPACGGQKQSPIDIQRRNVRHNPNMLQLELKGY : 73
ref|XP_013989824.1| : ---MECFSFFV---QLMLVSVASAGIDGIHWTYT-EGALLDQVHWAEHYPACGGRKQSPIDIQRQNVLHNPHMTRLELTGY : 73
ref|XP_014002952.1| : ---MECFSLFV---HFMLVSVASAGIDGIHWTYT-EGALLDQVHWAEEYPACGGRRQSPIDIQRRSVRHNPHMLQLELTGY : 73
ref|XP_007900354.1| : ----------------------MFQFTQGHWTYQ-EGDMDEEHWESQYPACAGKHQSPIDIQRKKTKYNPHLPQIQLSGY : 57
ref|NP_001085550.1| : MARLLHLGLYLFIVLSCH----LTNSHVVEWTYQ-EGELDEANWGKKYPTCAAKHQSPIDIQRKKVRHNPELTQLELIGY : 75
ref|XP_005986136.1| : ----------------------------------------MDEKHWSLKYPACSGKHQSPIDIQRKKVKYSPDFKKLVLNGY : 42
ref|XP_007432107.1| : ----MRTSGWVLQLCFLH----LSSARVLQWTYN--GERDEEHWGRYFENCLGKLQSPINIQRSKAKFNPDLELLELHGY : 70
ref|XP_006035521.2| : ----MRITGTVLHLLLMH----LSSSHITHWTYK-EGELDEEHWGRHFADCAGKHQSPIDIQKRKVRYNPQLMELELSGY : 71
ref|XP_014465858.1| : ----MRITGTVLHLLLMH----LSSSHITHWTYK-EGELDEEHWGRHFADCAGKHQSPIDIQKRKVRYNPQLMELELSGY : 71
ref|XP_005293083.1| : ----MRIPTTLLHLLLVH----LSASHTIHWTYK-AGELDEVHWGKHFAACAGKHQSPIDIQKRKVRYNPQLMQLELSGY : 71
ref|XP_006127158.1| : ----MRISTTLLHLLLVH----LSTSHVTQWTYK-ACALDEEHWGKHFADCAGKHQSPIDIQKRKVKYNPQLMQLELSGY : 71
ref|XP_010213863.1| : ------MRVPPAVLLLAR----LSCAHLAHWTYK-EGELDEEHWAKHFADCAERQSPIDIQKKKVVYNPDLAQLELRGY : 69
ref|XP_005057921.1| : -----MLMAVLLFLLLPP----LTHSHEVSWTYQGEGELDEEHWGQHFPACAGKQQSPIDIQRRKVRYNPL-LQLELTGY : 70
ref|XP_002187446.1| : ----MLMAALLLCLLLVP----LSHCHEVPWTYTGEGELDEEHWGQHFPDCAGQQQSPIDIQRRKVRYNPL-LQLELGGY : 71
gb|KFO64797.1|      : -----------------------------------GEGELDEEHWGQHFPDCAGKQQSPIDIQRRKVRYNPLLLQLELGGY : 46
ref|XP_425745.4|    : ------MLIPTALLLLQL----LSCSHAVHWTYR-EGELDEEHWGLHFAACAGKQQSPIDIQRRNVVHNAQLLQLELSGY : 69
ref|XP_010721064.1| : ------MLIPTALLLLQL----LSCSHAVHWTYR-EGELDEEHWGLHFAACAGKQQSPIDIQRKKVVHNVQLLQLELSGY : 69
gb|KFW85044.1|      : -----------------------------------EGELDEEHWGKHFPDCAGKSQSPVDIQRKKVRYNPLLEELELNGY : 45
ref|XP_013049674.1| : ---MRVPAAPLRLLLLLQ----LSCSHTVHWTYK-EGELDEEHWGQHFAACAGKRQSPIDIQRKKVVHNPQLLQLELSGY : 72
gb|KFQ22427.1|      : -----------------------------------EGELDEEHWAMHFADCAGKHQSPIDIQRKRVKYNPQLLQLELSGY : 45
ref|XP_005143337.1| : ---MWITA-ALFYLLLVH----PSCSHLVHWTYK-EGELDEEHWGKHFADCAGKRQSPVDIQRKKVRYNPQLLQLELSGY : 71
gb|KFQ51874.1|      : -----------------------------------EGELDEEHWGKHFADCAGKRQSPVDIQRKKVRYNPQLLQLELSGY : 45
gb|KFP87788.1|      : -----------------------------------EGELDEEHWGKHFPDCAGKHQSPIDIQRKKVRYNPQLLQLELNGY : 45
gb|KFP08430.1|      : -----------------------------------EGELDEEHWGEHFAECAGKHQSPIDIQRKKVRYNPQLLQLELSGY : 45
gb|KFP31739.1|      : -----------------------------------EGELDEEHWGKHYADCAGKQQSPIDIQRKKVRYNPQLLQLELSGY : 45
gb|EMC79327.1|      : ---MWIRA-VWFQLLLGH----LSCSHLPHWTYK-EGELDEEHWGKHFADCAGKHQSPIDIQRKKVTYNPQLLQLELSGY : 71
ref|XP_005237901.1| : ---MWITA-VLCHLLLVH----LSCSHLVHWTYK-EGELDEEHWGRHFADCAGKHQSPIDIQRKRVRYNPQLLRLELSGY : 71
gb|KFV72304.1|      : -----------------------------------EGELDEEHWGKHFADCAGKNQSPVDIQRKKVKYNPQLLQLELNGY : 45
gb|KFU95365.1|      : -----------------------------------EGELDEEHWGEHFPECAGKHQSPIDIQKKKVRYNPQLLQLELSGY : 45
gb|KFU99210.1|      : --------------------------------E--GELDEEHWGKHFTDCAGKHQSPIDIQRRKVRYNPQLLQLELTGY : 45
ref|XP_010076305.1| : ---MRITA-GWFPLLLVP----LGCSHDVHWTYT--GELDEEHWGKHFTDCAGKHQSPIDIQRRKVRYNPQLLQLELTGY : 70
gb|KFQ39105.1|      : ---------------------------LLLLPA-EGELDEEHWGKDFPDCAGKHQSPIDIQRKKVRYNPQLLQLELTGY : 51
gb|KFW07663.1|      : -----------------------------------EGELDEEHWGKHFADCAGRHQSPIDIQRKKAKYNLQ-LQLELSGY : 44
gb|KFO73096.1|      : -----------------------------------EGELDEEHWGKHFADCAGKRQSPIDIQRKKVMYNPQLLQLELSGY : 45
gb|KFV05980.1|      : -----------------------------------EGELDEEHWGKHFADCAGKHQSPIDIQRKKVRYNPQLLPLELSGY : 45
gb|KFO89464.1|      : --------------------------SSSLLLLA-EGELDEEHWGKHFADCAGQHQSPIDIQRKKVRYNPQLLQLELSGY : 54
gb|KFP34798.1|      : -----------------------------------EGELDEEHWGKHFADCAGKHQSPVDIQRKKVRYNPQLLQLELSGY : 45
gb|KFW93611.1|      : -----------------------------------EGELDEEHWGTHFADCAGKHQSPIDIQRKKVRYNPQLLQLELSGY : 45
gb|KFP22238.1|      : -----------------------------------EGELDEEHWGKHFADCAGQHQSPIDIQRKKVRYNPQLLQLELSGY : 45
gb|KFQ78929.1|      : -----------------------------------EGELDEEHWGRHFADCAGTQQSPIDIQRKKVRYNPQLLPLELRGY : 45
gb|KFP87033.1|      : -----------------------------------EGELDEEHWAKHFADCAGKHQSPVDIQRKKVRYNPQL-ELQLSGY : 44
gb|KGL96941.1|      : -----------------------------------EGDLDEEHWGTHFADCAGKHQSPIDIQRKKVRYNPQLLQLELSGY : 45
gb|KFZ60550.1|      : -----------------------------------EGELDEEHWGKHFTDCAGKHQSPIDIQRKKVRYNPQLLQLELSGY : 45
gb|KFP94842.1|      : -----------------------------------EGKLDEEHWGKHFADCAGKHQSPIDIQRKKVRYNPQLLQLELSGY : 45
ref|XP_011583597.1| : ---MWITA-VLFQLLLVH----LGCSHLLHWTYK-EGELDEEHWGKHFADCAGKHQSPIDIQRKKVRYNPQLLQLELSGY : 71
gb|KFZ62512.1|      : -----------------------------------EGELDEEHWGKHFADCAGKHQSPIDIQRKKVRYNPQLLQLELSGY : 45
gb|KFQ06064.1|      : -----------------------------------EGELDEEHWGKHFADCAGKRQSPIDIQRKKARYNPQLLQLELNGY : 45
ref|XP_009965819.1| : ---MWVTA-VLFYLLLVH----LSCSHLVHWTYK-EGELDEEHWGRHFADCAGKHQSPIDIQRKKVQYNPQLLQLELSGY : 71
gb|KFV47237.1|      : -----------------------------------EGELDEEHWGKRFADCAGKHQSPIDIQRKKVRYNPQLLQLELSGY : 45
gb|KFO11499.1|      : -----------------------------------EGELDEEHWGKHFADCAGKHQSPIDIQRKKVRYNPQLLQLELSGY : 45
gb|KFQ78854.1|      : -----------------------------------EGELDEEHWGKHFADCAGKHQSPIDIQRKKVRYNPQLLQLELSGY : 45
gb|KFW11285.1|      : -----------------------------------EGELDEEHWGKHFAGCAGKHQSPIDIQRKKVRYNPQLLQLELSGY : 45
gb|KFQ66673.1|      : -----------------------------------EGELDEEHWGKHFADCAGKHQSPIDIQRKKVRYNPQLLQLELSGY : 45
gb|KFQ93800.1|      : -----------------------------------EGELDEEHWGKHFADCAGKHQSPIDIQRKKVRYNPQLLQLELSGY : 45
gb|KFP53434.1|      : -----------------------------------EGELDEEHWGKHFADCAGKHQSPIDIQRKKVRYNPQLLQLELSGY : 45
ref|XP_009323546.1| : ---MWVPA-VLFRLLLVH----LSCSHLVHWTYK-EGELDEEHWGKHFADCAGKHQSPIDIQRKKVRYNPQLLQLELSGY : 71
gb|KFM01971.1|      : -----------------------------------EGDLDEEHWGKHFADCAGKHQSPIDIQRKKVRYNPQLLQLELSGY : 45
                                                           eg ld  hW      C gk QSPidIQr  v  np  lqlel gY
```

```
                                    *       100         *       120         *       140         *       160
ref|XP_012726090.1| : DAQ-KGNFRMTNNGHTVQIDLPPSMKITKGLPNEFTAVQLHLHWGGQDQEESGAEHTIDGVRYMAELHIVHYNSGKYKNF : 153
ref|XP_007570993.1| : ETQ-KGNFRMTNNGHTVQIDLPPSMKITKGLPGQFTAVQMHLHWGGLDLEESGAEHTIDGSRYMAELHVVHYNSDKYKSF : 153
ref|XP_008411549.1| : DAQ-KGNFRMINNGHTVQIDLPPSMKITKGLPGQFTAVQMHLHWGGLDLEESGAEHTIDGSRYMAELHVVHYNSDKYKSF : 153
ref|XP_004070439.1| : DAQ-QGKFLMTNNGHTVQINLPPTMTISQGLPGIYTAVQMHLHWGGSHQEASGAEHTIDGVRYMAELHIVHYNSEKYRSF : 151
emb|CAF93821.1|     : DAQ-QGTFLMTNNGHTVQIDLPPTMVITEGLPGKYTAVQMHLHWGGWDLEASGAEHTIDGVRYMAELHVVHYNSDKYKSF : 153
ref|XP_011620264.1| : NAQ-RGDFLMTNNGHTVQIDLPPTMMITEGLPGKYTAVQMHLHWGGWDLEASGAEHTIDGIRYMAELHVVHYNSDKYKSF : 154
ref|XP_013875818.1| : DHQ-KGTFLMSNNGHSVQIDLPPTMMITQGLPGKYTAVQMHLHWGGWDLEASGAEHTIDGIRYMAELHVVHYNSDKYTSF : 155
ref|XP_005478239.2| : DAQ-TGTFLMSNNGHSVQIELPPSMKITEGLPGKYTAVQMHLHWGGWDLEASGAEHTIDGIRYMAELHVVHYNSDKYKSF : 154
ref|XP_006785611.1| : DAQ-KGTFLMSNNGHSVQIDLPPSMKITEGLPGKYTAVQMHLHWGGWDLEASGAEHTIDGIRYMAELHVVHYNSDKYKSF : 154
ref|XP_005914985.1| : DAQ-KGTFLMSNNGHSVQIDLPPSMKIIEGLPGKYTAVQMHLHWGGWDLEASGAEHTIDGIRYMAELHVVHYNSDKYKSF : 154
ref|XP_004554282.1| : DAQ-KGTFLMSNNGHSVQIDLPPSMKITEGLPGKYTAVQMHLHWGGWDLEASGAEHTIDGIRYMAELHVVHYNSDKYKSF : 154
ref|XP_005733115.1| : DAQ-KGTFLMSNNGHSVQIDLPPSMKIIEGLPGKYTAVQMHLHWGGWDLEASGAEHTIDGIRYMAELHVVHYNSDKYKSF : 154
ref|XP_010739228.1| : DAQ-KGTFLMSNNGHSVQIDLPPTMVITKGLPGKYTAVQMHLHWGGWDLEASGAEHTVDGIRYMAELHVVHYNSDKYNSF : 153
ref|XP_008317045.1| : DTQ-RGTFRMSNNGHTVQIDLPPTMMITKGLPGKYTAVQMHLHWGGWDLEASGAEHTIDGIRYMAELHVVHYNSDKYKSF : 154
ref|XP_010793030.1| : DAQ-SGNFLMSNNGHSVQIDLPPTMMITKGLPGKYTAVQMHLHWGGWDLEASGAEHTIDGIRYMAELHVVHYNSDKYKSF : 155
ref|XP_008294025.1| : DAQ-MGNFLMSNNGHSVQIDLPPSMMITKGLPGKYTAVQMHLHWGGWDLEASGAEHTIDGIRYMAELHVVHYNSDKYKSF : 154
ref|XP_002666525.1| : EDI-RGSFLMKNNGHSVEIQLPSTMKITKGFPHQYTAVQMHLHWGGWDLEASGSEHTMDGIRYMAELHVVHYNSEKYPSF : 152
ref|XP_009295179.1| : EDI-RGSFLMKNNGHSVEIQLPSTMKITKGFPHQYTAVQMHLHWGGWDLEASGSEHTMDGIRYMAELHVVHYNSEKYPSF : 152
ref|XP_007246352.1| : GEVKNGNFLMTNNGHSVQINLPPSMKITKGLPSRYTAVQMHLHWGGWDLEASGAEHTLDGIRYMAELHVVHFNSEKYKTF : 150
gb|KPP63671.1|      : DQQ-KGNFLMTNNGHSVQINLPPTMMITKGLPARYTAVQMHLHWGGWDLEASGAEHTLDGIRYMAELHVVHYNSEKYKSF : 118
ref|XP_012681508.1| : DEI-NGKFLMTNNGHSVQIDLPPTMMITEGLPDRYTAVQMHLHWGGWDLEASGAEHTVDGIRYMAELHVVHYNSEKYKSF : 121
ref|XP_010902628.1| : GPM-KGNFLMTNNGHSVQIDLPPSMVITNGLPNKYTAVQMHLHWGGWDLEESGAEHTLDGIRYMAELHVVHYNSEKYSSF : 152
ref|XP_013989824.1| : DAQ-RGNFLMKNNGHSVEIVLPPSMVITKGLPGRYTAVQMHLHWGGWDLEESGSEHTLDGIRYMAELHVVHYNSDKYKSF : 152
ref|XP_014002952.1| : DAQ-KGNFLMKNNGHSVEIVLPPSMVITKGLPGGYTAVQMHLHWGGWDLEESGAEHTLDGIRYMAELHVVHYNSDKYKSF : 152
ref|XP_007900354.1| : DAHYEEPLRLTNNGHSVALGVPPNLFISHGLQNKFTAVQMHFHWGGLDLESSGSEHTVDGMRYIAELHIVHYNSEKYKTF : 137
ref|NP_001085550.1| : DGPLTGHFKVTNNGHSVQIDLPSTMTIKKGLNSLYTAVQMHLHWGGLESETSGSEHTIDGMRYLAELHIVHYNSGAYKSF : 155
ref|XP_005986136.1| : DGSQNGAFKMTNNGHSVQIDLPPTMNISEGFTSNYTAVQMHFHWGGLEQESSGSEHTVDGMRYLAELHIVHYNSDKYSKF : 122
ref|XP_007432107.1| : GHR-HDNFRMTNNGHSVVIQLPSSMIIMKGLPGKFTAIQLHLHWGGMDLESSGSEHTIDGMRYMAELHIVHYNSEAYSSF : 149
ref|XP_006035521.2| : DGPLQGNFTMTNNGHSVQIDLPPTMRITKGLPSRFTAVQMHLHWGGLDLETSGSEHTIDGMRYFAELHIVHYNSGNYSSF : 151
ref|XP_014465858.1| : DGPLQGNFTMTNNGHSVQIDLPPTMRITKGLPSLFTAVQMHLHWGGLDLETSGSEHTIDGMRYFAELHIVHYNSGNYSSF : 151
ref|XP_005293083.1| : DGPLQGNFTMTNNGHSVQIDLPPTMRITKGLPSLFTAVQMHLHWGGLDLETSGSEHTVDGMRYFAELHIVHYNSGNYSSF : 151
ref|XP_006127158.1| : DGPLQGNFTMTNNGHSVQIDLPPTMRITKGLSSIYTAVQMHLHWGGLDLETSGSEHTMDGMRYLAELHVVHYNSGNYSSF : 151
ref|XP_010213863.1| : DGPLQGSFTMTNNGHSVQIDLPPSLRVSVGLAGPYTAVQMHLHWGGLDLESSGSEHTVDGMRYLAELHVVHYNSANYSSF : 149
ref|XP_005057921.1| : EGPLQGHFRMTNNGHSVQIDLPPTMSISRGLPGLYRAVQMHLHWGGLELESSGSEHTIDGMRHFAELHIVHYNSANYSSF : 150
ref|XP_002187446.1| : EGPLQGQLRMTNNGHSVQIDLPPTMNISRGLPGVYTAVQMHLHWGGLDLESSGSEHTIDGMRYFAELHIVHYDSANYSSF : 151
gb|KFO64797.1|      : DGPLQGQFRMTNNGHSVQIDLPPTMNISRGLPGVYTAVQMHLHWGGLDLETSGSEHTIDGMRYFAELHIVHYNSANYSSF : 126
ref|XP_425745.4|    : SRPLQEDCTMTNNGHSVQIDLPPTLSISRGLPRPYTAVQMHLHWGGLDLETSGSEHTLDGMRYVAELHIVHYNSADYSSF : 149
ref|XP_010721064.1| : SRPLQGDFTMTNNGHSVQIDLPPTLSISRGLPHPYTAVQMHLHWGGLDLETSGSEHTLDGMRYVAELHIVHYNSADYSSF : 149
gb|KFW85044.1|      : DGPLQGDFKMTNNGHSVQIDLPPTMNISRGLPGLYTAVQMHLHWGGLDLETSGSEHTIDGLRYFAELHIVHYNSADYSSF : 125
ref|XP_013049674.1| : DEPLQGDFTMTNNGHSVQIDLPPTLHIARGLPSLYTAVQMHLHWGGLDLETSGSEHTLDGMRYVAELHIVHYNSAEYSSF : 152
gb|KFQ22427.1|      : DGPLQGDLTMTNNGHSVQIDLPPTMHISRGLPGLYTAVQMHLHWGGLDLETSGSEHTIDGMRYLAELHIVHYNSANYSSF : 125
ref|XP_005143337.1| : DGPLKGDFTMTNNGHSVQIDLPPTMHISRGLPGLYTAVQMHLHWGGLDLESSGSEHTIDGMRYFAELHIVHYNSAEYSSF : 151
gb|KFQ51874.1|      : DGPLQGDFTMTNNGHSVQIDLPPTMHISRGLPGLYTAVQMHLHWGGLDLETSGSEHTIDGMRYFAELHIVHYNSADYLSF : 125
gb|KFP87788.1|      : DGPLQGDFTMSNNGHSVQINLPPTMNISRGLPGLYTAVQMHLHWGGLDLETSGSEHTIDGMRYFAELHIVHYNSADYSSF : 125
gb|KFP08430.1|      : DGPLQGDFTMTNNGHSVQIDLPPTMHISRGLPGLYTAVQMHLHWGGLDLETSGSEHTIDGMRYFAELHIVHYNSADYSSF : 125
gb|KFP31739.1|      : DGPLQGNFTMTNNGHSVQIDLPPSMHISQGLPGLYTAVQMHLHWGGLDLETSGSEHTIDGMRYFAELHIVHYNSADYSSF : 125
gb|EMC79327.1|      : DGPLQGDFTMTNNGHSVQIDLPPTMYISRGLPGLYTAVQMHLHWGGLDLETSGSEHTIDGMRYFAELHIVHYNSAAYSSF : 151
ref|XP_005237901.1| : DGPLQGDFTMTNNGHSVQIDLPPTMRISRGLPGVYTAVQMHLHWGGLDLETSGSEHTIDGMRYLAELHIVHYNSADYSSF : 151
gb|KFV72304.1|      : DGPLQGDFTMTNNGHSVQIDLPPTMHISRGLPSLYTAVQMHLHWGGLDLETSGSEHTIDGMRYFAELHIVHYNSADYLSF : 125
gb|KFU95365.1|      : DGPLQGDFMMTNNGHSVQIDLPPTMHISRGLPGLYTAVQMHLHWGGLDLETSGSEHTIDGMRYFAELHIVHYNAADYSSF : 125
gb|KFU99210.1|      : DGPLQGDFTMTNNGHSVQIDLPPTMHISGGLPGPSPAVQMHLHWGGLDLETSGSEHTLDGMRYFAELHIVHYNSADYSSF : 125
ref|XP_010076305.1| : DGPLQGDFTMTNNGHSVQIDLPPTMHISGGLPGPSPAVQMHLHWGGLDLETSGSEHTLDGMRYFAELHIVHYNSADYSSF : 150
gb|KFQ39105.1|      : DGPLQGDFIMTNNGHSVQIDLPPTMNISRGLPGLYTAVQMHLHWGGLDLETSGSEHTVDGMRYFAELHIVHYNSADYSSF : 131
gb|KFW07663.1|      : DGPLQGEFTMTNNGHSVQIDLPPTMHISRGLPGRYAAVQMHLHWGGLDLETSGSEHTIDGMRYFAELHIVHYNSANYSSF : 124
gb|KFO73096.1|      : DGPLQGDFTMTNNGHSVQFDLPPTMHISRGLPGLYTAVQMHLHWGGLDLETSGSEHTINGMRYFAELHIVHYNSAEYSSF : 125
gb|KFV05980.1|      : DEPSQGDFTMTNNGHSVQIDLPPTMHISRGLPSLYTAVQMHLHWGGLDLETSGSEHTIDGMRYFAELHIVHYNSADYPSF : 125
gb|KFO89464.1|      : DGPLQGDFTMTNNGHSVQIDLPPTMYITRGLPGLYTAVQMHLHWGGLELETSGSEHTIDGMRYFAELHIVHYNSANYSSF : 134
gb|KFP34798.1|      : DGPLQGDFTMTNNGHSVQIDLPPTMHISRGLPALYTAVQMHLHWGGLDLETSGSEHTIDGMRYFAELHIVHYNSADYSSF : 125
gb|KFW93611.1|      : DGPLRGDFTMTNNGHSVQIDLPPTMHISGGLPSLYTAVQMHLHWGGLDLETSGSEHTIDGMRYFAELHIVHYNSADYSSF : 125
gb|KFP22238.1|      : DGPLQGDFTMTNNGHSVQIHLPPTMHISRGLPGLYTAVQMHLHWGGLDLETSGSEHTIDGMRYFAELHIVHFNSANYSSF : 125
gb|KFQ78929.1|      : DGPLQGDFAMTNSGHSVQIDLPPTMHISGGLPGLYTAVQMHLHWGGLDLETSGSEHTIDGMRYFAEMHIVHYNSADYSSF : 125
gb|KFP87033.1|      : DGPLQGNFTMTNNGHSVQIDLPPTMHISRGLPGLYTAVQMHLHWGGLDLETSGSEHTVDGMRYFAELHIVHYNSADYSSF : 124
gb|KGL96941.1|      : DGPLRGDFTMTNNGHSVQIDLPPTMRISRGLPGIYTAVQMHLHWGGLDLETSGSEHTIDGMRYFAELHIVHYNSADYSSF : 125
gb|KFZ60550.1|      : DGPLEGDFTMTNNGHSVQIDLPPTMHISRGLPGLYTAVQMHLHWGGLDLETSGSEHTIDGMRYFAELHIVHYNSADYSSF : 125
gb|KFP94842.1|      : DGPLQGDFTMTNNGHSVQIDLPPTMHISGGLPGLYTAVQMHLHWGGLDLETSGSEHTIDGMRYFAELHIVHYNSADYSSF : 125
ref|XP_011583597.1| : DGPLQGDFTMTNNGHSVQIDLPPTMHISGGLPGLYTAVQMHLHWGGLDLETSGSEHTIDGMRYFAELHIVHYNSADYSSF : 151
gb|KFZ62512.1|      : DGPLQGDFTMTNNGHSVQIDLPPTMHISRGLPGLYTAVQMHLHWGGLDLETSGSEHTIDGMRYFAELHIVHYNSADYSSF : 125
gb|KFQ06064.1|      : DGPLQGDFTMTNNGHSVQIDLPPTMHISRGLPGLYTAVQMHLHWGGLDLETSGSEHTIDGMRYFAELHIVHYNSADYSSF : 125
ref|XP_009965819.1| : DGPLQGDFTMTNNGHSVQIDLPPTLHISRGLPGLYTAVQMHLHWGGLDLETSGSEHTIDGMRYFAELHIVHYNSADYSSF : 151
gb|KFV47237.1|      : DGPLQGDFTMTNNGHSVQIDLPPTMHISRGLPGLYTAVQMHLHWGGLDLETSGSEHTIDGMRYFAELHIVHYNSADYSSF : 125
gb|KFO11499.1|      : DGPLQGDFTMTNNGHSVQIDLPPTMHISRGLPGFYTAVQMHLHWGGLDLETSGSEHTIDGMRYFAELHIVHYNSADYSSF : 125
gb|KFQ78854.1|      : DGPLQGDFTMTNNGHSVQIDLPPTMHISRGLPGLYTAVQMHLHWGGLDLETSGSEHTIDGMRYFAELHIVHYNSADYSSF : 125
gb|KFW11285.1|      : DGPLQGDFTMTNNGHSVQIDLPPTMHISRGLPGLYTAVQMHLHWGGLDLETSGSEHTIDGMRYFAELHIVHYNSADYSSF : 125
gb|KFQ66673.1|      : DGPSQGDFTMTNNGHSVQIDLPPTMHISRGLPSLYTAVQMHLHWGGLDLETSGSEHTVDGMRYFAELHIVHYNSADYSSF : 125
gb|KFQ93800.1|      : DGPLQGDFTMTNNGHSVQIDLPPTMHISRGLPGLYTAVQMHLHWGGLDLETSGSEHTIDGMRYFAELHIVHYNSADYSSF : 125
gb|KFP53434.1|      : DGPLQGDFTMTNNGHSVQIDLPPTMHISRGLPGLYTAVQMHLHWGGLDLETSGSEHTIDGMRYFAELHIVHYNSADYSSF : 125
ref|XP_009323546.1| : DGPLQGDFTMTNNGHSVQIDLPPTMHISRGLPGLYTAVQMHLHWGGLDLETSGSEHTIDGMRYFAELHIVHYNSADYSSF : 151
gb|KFM01971.1|      : DGPLQGDFTMTNNGHSVQIDLPPTMHISRGLPGLYTAVQMHLHWGGLDLETSGSEHTIDGMRYFAELHIVHYNSADYSSF : 125
                      d    g f mtNnGHsVqidlPp m i  Glp  ytAvQmHlHWGG dlE SG EHT dG Ry AElH VHyns  Y sF
```

```
                          *        180         *        200         *        220         *        240
ref|XP_012726090.1| : TEAQDKHDGLAVLAFVYDDSHYENTYYSDFISNLGKIKFAGQSMYISDINVRSMLPENLNDFFRYEGSLTTPPCHESIIW : 233
ref|XP_007570993.1| : SEAKDKSDGLAVLAFVYDYGHFENTYYSDFISNLEKIKYAGQSMNISSIAVRDMLPENLSHFFRYEGSLTTPPCFESIIW : 233
ref|XP_008411549.1| : SEAKDKSDGLAVLAFVYDYGHFENTYYSDFISNLEKIKYAGQSMNISSIDVLSMLSENLSHFFRYEGSLTTPPCFESIIW : 233
ref|XP_004070439.1| : SEAKDKKDGLAVLAFVYDDGHFENTYYSDFIANLEKIKYAGQSMNITSIDVRSMLPENLNHFFRYEGSLTTPPCFESIIW : 231
emb|CAF93821.1|     : IEARDKPDGLAVLAFFYDDGHFENTYYSDFIANLGKIKYAGQSMYISSLDVRSMLPENLNHFFRYQGSLTTPPCYESILW : 233
ref|XP_011620264.1| : IEARDKPDGLAVLAFFYDDGHFENTYYSDFISNLGKIKYAGQSMNISSLDMRSMLPENLNHFFRYQGSLTTPPCYESILW : 234
ref|XP_013875818.1| : SEAKDKPGGLAVLAFFYEDGHFENTYYSDFISNLAKIQFAGQTMNISNIDVRSMLPENLNHFFRYEGSLTTPPCFESIIW : 235
ref|XP_005478239.2| : TEARDKPDGLAVLAFFYEDGHFENTYYSDFIANLEKIKYAGQSMNIHHLDVRSMLPENLNHFFRYQGSLTTPPCYESILW : 234
ref|XP_006785611.1| : TEARDKPDGLAVLAFFYEDGHFENTYYSDFIANLKKIKYAGQSMNISDLDVRSMLPENLNHFFRYQGSLTTPPCYESILW : 234
ref|XP_005914985.1| : TEARDKPDGLAVLAFFYEDGHFENTYYSDFIANLKKIKYAGQSMNISNLDVRSMLPENLNHFFRYQGSLTTPPCYESILW : 234
ref|XP_004554282.1| : TEARDKPDGLAVLAFFYEDGHFENTYYSDFIANLKKIKYAGQSMNISNLDVRSMLPENLNHFFRYQGSLTTPPCYESILW : 234
ref|XP_005733115.1| : TEARDKPDGLAVLAFFYEDGHFENTYYSDFIANLKKIKYAGQSMNISNLDVRSMLPENLNHFFRYQGSLTTPPCYESILW : 234
ref|XP_010739228.1| : TEARDKPDGLAVLAFFYDDGHFENTYYSDFISTLDKIKYAGQSMNISNIDVRSMLPENLNHFFRYQGSLTTPPCYESILW : 233
ref|XP_008317045.1| : SEAKDKPDGLAVLAFFYDDGHFENTYYSDFISNLDKIKYVGQSMNISSINVRSMLPENMNHFFRYQGSLTTPPCYESILW : 234
ref|XP_010793030.1| : TEARDKTDGLAVLAFFYDDGHFENTYYSDFISNLDNIKYTGQSMNISGIDVRSMLPENLNHFFRYEGSLTTPPCYESILW : 235
ref|XP_008294025.1| : TEARDKPDGLAVLAFFYDDGHFENTYYSDFISNLDRIKYAGQSMNISSINVRSMLPENLNHFFRYEGSLTTPPCYESILW : 234
ref|XP_002666525.1| : EEAKNKPDGLAVLAFFFEDGHFENTYYSDFISNLANIKYVGQSMSISNLNVLSMLSENLSHFYRYKGSLTTPPCFESVMW : 232
ref|XP_009295179.1| : EEAKNKPDGLAVLAFFFEDGHFENTYYSDFISNLANIKYVGQSMSISNLNVLSMLSENLSHFYRYKGSLTTPPCFESVMW : 232
ref|XP_007246352.1| : EEAVDKPDGLAVLAFFYEDGHFENTYYSEFINNLAKVRYAGQSMNISSLNVRSMLPENLANFFRYEGSLTTPPCFESILW : 230
gb|KPP63671.1|      : DEAVDKPDGLAVLAFFYEDGHFENTYYSDFISNLAKVKFAGQSMNISTINVRSMLPENLSQFYRYQGSLTTPPCYESILW : 198
ref|XP_012681508.1| : AEAKDKSDGLAVLAFFYEDGHFENTYYSDFISNLAKVKYAGQSMNLSTINVRSMLPENMNHFFRYEGSLTTPPCYESIIW : 201
ref|XP_010902628.1| : DEARNKTDGLAVLAFFYEDGHFENTYYSDFINNLAKVKYAKQSMNISTLNVRSMLPENLNQFFRYQGSLTTPPCYESVLW : 232
ref|XP_013989824.1| : QEASNKPDGLAVLAFFFEDGHFENTYYSDFINNLEKVKYTGQSLNISTLNVRSMLPENLKHFYRYQGSLTTPPCYESILW : 232
ref|XP_014002952.1| : QEASDKPDGLAVLAFFYEDGHFENTYYSDFISNLGKVKYAGQSMNISTLNVRSMLPENLNHFFRYQGSLTTPPCYESILW : 232
ref|XP_007900354.1| : EEAHDKEDGLAVLAFFYEDGHFENTYYSDFISSLGKVRYAGQTTQMHHLDISTMLSDDLSQFYRYKGSLTTPPCFESITW : 217
ref|NP_001085550.1| : DEAKDKPNGLAVLAFLYTTGNYENTYYSDFISKLAKIRYAGQETEMHTLDVMAMLPENLVNFYRYDGSLTTPPCTENVLW : 235
ref|XP_005986136.1| : DDAKDKPDGLAVLAFFYEDGHFENTYYTDFISHLAKIRFAGQYTHLTLINVRDMLPEDLSHFYRYKGSLTTPPCYESITW : 202
ref|XP_007432107.1| : EEAKDKPDGLAVLAFLFVGTHFENTYYSDFITKLDKVKFAGESTILHSLDILAMLPDNLSNFYRYHGSLTTPPCTENVIW : 229
ref|XP_006035521.2| : EEAKDKPNGLAVLAFLYVDGHFENTYYSKFISNLAKIRYAGQSMRLTSLDVQGMLPENLSHFYRYPGSLTTPPCSESVIW : 231
ref|XP_014465858.1| : EEAKDKPNGLAVLAFLYVDGHFENTYYSKFISNLAKIRYAGQSMRLTSLDVQGMLPENLSHFYRYPGSLTTPPCSESVIW : 231
ref|XP_005293083.1| : EEAKDKLNGLAVLAFLYVDGNFENTYYSEFISNLAKIRFAGQSTKLSLLDVQAMLPENLSHFYRYQGSLTTPPCSESVIW : 231
ref|XP_006127158.1| : EEAKDKRNGLAVLAFLYVDGNFENTYYSDFISKLAKIRFAGQSTKLSSLDVQAMLPENLSHFYRYHGSLTTPPCSESVIW : 231
ref|XP_010213863.1| : EEAKDKPNGLAVLAFLYVDGHFENTYYSEFISKLAQIRFAGQSTQLASLDVRAMLPENLSRFYRYQGSLTTPPCSESVTW : 229
ref|XP_005057921.1| : EEAKDKPQGLAVLAFLYTDGHFENTYYSEFISKLARIRFAGQSTTLSSLDVQAMLPENLSQFYRYQGSLTTPPCSESVIW : 230
ref|XP_002187446.1| : EEAKDKPQGLAVLAFLYTDGHFENTYYSEFISKLARIRFAGQSTTLSSLDIQAMLPENLSHFYRYQGSLTTPPCSESVIW : 231
gb|KFO64797.1|      : EEAKDKPQGLAVLAFLYSDGHFENTYYSEFISKLARIRFAGQSTMLSSLDVQAMLPENLAHFYRYQGSLTTPPCSESVIW : 206
ref|XP_425745.4|    : EEAKDKPNGLAVLAFLYVEGHLENTYYSEFISKLAKIRFAGQSTKLTSLDVQAMLPENLSHFYRYQGSLTTPPCSESVTW : 229
ref|XP_010721064.1| : EEAKDKPNGLAVLAFLYVEGHLENTYYSEFISKLAKIRFAGQSTKLTSLDVQAMLPENLSHFYRYQGSLTTPPCSESVTW : 229
gb|KFW85044.1|      : EEAKDKPNGLAVLAFLYEDGHFENTYYSEFISKLARIRFAGQSTKLSSLDVQAMLPENLSHFYRYQGSLTTPPCSESVIW : 205
ref|XP_013049674.1| : EDAKDKPNGLAVLAFLYAEGHFENTYYSEFISKLAKIKFAGQSTKLTSLDVQAMLPENLLHFYRYQGSLTTPPCSESVTW : 232
gb|KFQ22427.1|      : EEAKDKPNGLAVLAFLYTDGHFENTYYSEFISKLAKIRFAGQSTKLISLDVQAMLPENLSHFYRYQGSLTTPPCSESVIW : 205
ref|XP_005143337.1| : EEAKDKPNGLAVLAFLYADGHFENTYYSEFISKLAKIRFAGQSTKLLSLDVQAMLPENLSYFYRYQGSLTTPPCTESVIW : 231
gb|KFQ51874.1|      : EEAKDKPNGLAVLAFLYADGHFENTYYTEFISKLAKIRFAGQSTKLISLDIQAMLPENLSHFYRYQGSLTTPPCSESVIW : 205
gb|KFP87788.1|      : EEAKDKPNGLAVLAFLYTDGHFENTYYSEFISKLARIRFAGQSTKLNSLDVQAMLPENLSHFYRYQGSLTTPPCSESVIW : 205
gb|KFP08430.1|      : EEAKDKPNGLAVLAFLYVDGHLENTYYSEFISKLAKIRFAGQSTKLTSLDVQAMLPENLSHFYRYQGSLTTPPCFESVTW : 205
gb|KFP31739.1|      : EEAKDKPNGLAVLAFLYVDGHLENTYYSEFISKLAKIRFAGQSTKLISLDVQAMLPENLSHFYRYQGSLTTPPCSESVIW : 205
gb|EMC79327.1|      : EEAKDKPNGLAVLAFLYADGHFENTYYSEFISKLAKIRFAGQSTKLISLDVQAMLPENLSHFYRYQGSLTTPPCSESVIW : 231
ref|XP_005237901.1| : EEAKDKPNGLAVLAFLYTDGHFENTYYSELISKLAKIRFAGQSTKLFSLNVQAMLPENLSHFYRYQGSLTTPPCSESVLW : 231
gb|KFV72304.1|      : EEAKDKPNGLAVLAFLYVDGHLENTYYSEFISKLAKIRFAGQSTKLGSLDVQAMLPENLSHFYRYQGSLTTPPCSESVIW : 205
gb|KFU95365.1|      : EEAKDKPNGLAVLAFLYVDGHFENTYYSEFISKLAKIRFAGQSTKLISLDVQAMLPENLSHFYRYQGSLTTPPCSESVIW : 205
gb|KFU99210.1|      : EEAKDKPNGLAVLAFLYADGHFENTYYSEFISKLAKIRFAGQSTKLSSLDIQAMLPENLSHFYRYQGSLTTPPCSESIIW : 205
ref|XP_010076305.1| : EEAKDKPNGLAVLAFLYADGHFENTYYSEFISKLAKIRFAGQSTKLSSLDIQAMLPENLSHFYRYQGSLTTPPCSESIIW : 230
gb|KFQ39105.1|      : EEAKDKPNGLAVLAFLYVDGHFENTYYSEFISKLAKIRFAGQSTKLISLDIQAMLPENLSHFYRYQGSLTTPPCSESVIW : 211
gb|KFW07663.1|      : EEAKDKPNGLAVLAFLYTDGHFENTYYSEFISKLAKIKFAGQSTKLTSLDVQAMLPENLSYFYRYQGSLTTPPCSESVIW : 204
gb|KFO73096.1|      : EEAKDKPNGLAVLAFLYADGHFENTYYSEFISKLAKIRFAGQSTKLLSLDVRAMLPENLSHFYRYQGSLTTPPCSESVIW : 205
gb|KFV05980.1|      : EEAKDKPNGLAVLAFLYADGHFENTYYSEFISKLAKIRFAGQSTKLTSLDVQAMLPDNLSHFYRYQGSLTTPPCSESVIW : 205
gb|KFO89464.1|      : EEAKDKPNGLAVLAFLYADGHFENTYYSEFISKLAKIRFAGQSTKLISLDVQAMLPENLSHFYRYQGSLTTPPCSENVIW : 214
gb|KFP34798.1|      : EEAKDKPNGLAVLAFLYADGHFENTYYSEFISKLAKIRFAGQSTKLLSLDVQAMLPENLSHFYRYQGSLTTPPCSESVIW : 205
gb|KFW93611.1|      : EEAKDKPDGLAVLAFLYVDGHFENTYYSEFISKLAKIRFAGQSTKLVSLDVQAMLPENLSHFYRYHGSLTTPPCSESVIW : 205
gb|KFP22238.1|      : EEAKDKPNGLAVLAFLYVDGHFENTYYSEFISKLAKIRFAGQSTKLSSLDVQAMLPENLSHFYRYQGSLTTPPCSESVIW : 205
gb|KFQ78929.1|      : EEAKDKPNGLAVLAFLYADGHFENTYYSEFISKLAKVRFAGQSTKLISLDVQAMLPENLSHFYRYQGSLTTPPCSESVIW : 205
gb|KFP87033.1|      : EEAKDKPNGLAVLAFLYVDGHFENTYYSEFISKLAKIRFAGQSTKLISLDVQAMLPENLSHFYRYQGSLTTPPCSESVIW : 204
gb|KGL96941.1|      : EEAKDKPNGLAVLAFLYADGHFENTYYSEFISKLAKIRFAGQSTKLVSLDVQAMLPENLSHFYRYQGSLTTPPCSESVIW : 205
gb|KFZ60550.1|      : EEAKDKPNGLAVLAFLYADGHFENTYYSEFISKLARIRFAGQSTKLISLDVQAMLPENLSHFYRYQGSLTTPPCSESVIW : 205
gb|KFP94842.1|      : EEAKDKPNGLAVLAFLYVDGHFENTYYSEFISKLAKIRFAGQSTKLISLDVQAMLPENLSHFYRYQGSLTTPPCFESVIW : 205
ref|XP_011583597.1| : EEAKDKPNGLAVLAFLYVDGHFENTYYSEFISKLAKIRFAGQSTKLISLDVQAMLPENLSHFYRYQGSLTTPPCFESVIW : 231
gb|KFZ62512.1|      : EEAKDKPNGLAVLAFLYADGHFENTYYSEFISKLAKIRFAGQSTKLISLDVQAMLPENLLHFYRYQGSLTTPPCSESVLW : 205
gb|KFQ06064.1|      : EEAKDKPNGLAVLAFLYTDGHFENTYYSEFISKLAKIRFAGQSTKLISLDVQAMLPENLSHFYRYQGSLTTPPCSESVIW : 205
ref|XP_009965819.1| : EEAKDKPNGLAVLAFLYADGHFENTYYSEFISKLAKIRFAGQSTKLISLDVQAMLPENLSHFYRYQGSLTTPPCSESVIW : 231
gb|KFV47237.1|      : EEAKDKPNGLAVLAFLYTDGHFENTYYSEFISKLAKITFAGQSTKLNSLDVQAMLPENLSHFYRYQGSLTTPPCSESVIW : 205
gb|KFO11499.1|      : EEAKDKPNGLAVLAFLYADGHFENTYYSEFISKLAKIRFAGQSTKLISLDVQAMLPENLSHFYRYQGSLTTPPCSESVIW : 205
gb|KFQ78854.1|      : EEAKDKPNGLAVLAFLYADGHFENTYYSEFISKLAKVRFAGQSTNLVSLDVQAMLPENLSHFYRYQGSLTTPPCSESVIW : 205
gb|KFW11285.1|      : EEAKDKPHGLAVLAFLYADGHFENTYYSEFISKLAKIRFAGQSTKLLSLDVQAMLPENLSHFYRYQGSLTTPPCSESVIW : 205
gb|KFQ66673.1|      : DEAKDKPNGLAVLAFLYVDGHFENTYYSEFISKLAKIRFAGQSTKLSSLDVQAMLPENLSHFYRYQGSLTTPPCSESVIW : 205
gb|KFQ93800.1|      : EEAKDKPNGLAVLAFLYVDGHFENTYYSDFISKLAKIRFAGQSTKLISLDVQAMLPENLSHFYRYQGSLTTPPCSESVIW : 205
gb|KFP53434.1|      : EEAKDKPNGLAVLAFLYADGHFENTYYSEFISKLAKIRFAGQSTKLISLDVQAMLPENLSRFYRYQGSLTTPPCSESVIW : 205
ref|XP_009323546.1| : EEAKDKPNGLAVLAFLYVDGHFENTYYSEFISKLAKIRFAGQSTKLISLDVQAMLPENLSHFYRYQGSLTTPPCSESVIW : 231
gb|KFM01971.1|      : KEAKDKPNGLAVLAFLYADGHFENTYYSEFISKLAKIRFAGQSTKLISLDVQAMLPENLSHFYRYQGSLTTPPCSESVIW : 205
                       eA dKp GLAVLAF y dghfENTYYs fIs L ki  agqs     ldv  MLpenl hF RY GSLTTPPC Es  W
```

```
                            *        260         *        280         *        300         *        320
ref|XP_012726090.1| : TVFDTPITLSDNQITKLERSIMDLDNKTLWNDYRIAQPLNDRVVESSFRQK------KSCLQDVIEYRFQKIEGLITSLG : 307
ref|XP_007570993.1| : TVFDTPISLSHNQIRKLESTIMDIDNKTLWNDYRIAQPLNDRVVESSFMPRLGK--GTFCQQDEIESKILKIEGLITSLG : 311
ref|XP_008411549.1| : TVFDTPITLSHNQIRKLESTIMDVDNKTLWNDYRIAQPLNDRVVESSFMPRLGK--GTFCQQDEIESKILKIEGLITSLG : 311
ref|XP_004070439.1| : TVFDTPITLSQNQIRRLESTIMDIDNKTLWNDYRIAQPLNDRVVESSFLPRVRK--GSFCLQDVIESRIEKIEGLITSLG : 309
emb|CAF93821.1|     : TVFDTPITLSHNQIRKLESTIMDLDNKTLWNDYRIAQPLNDRVVESSFLPRLGK--GTFCRQDEIESKILKIEGLITSLG : 311
ref|XP_011620264.1| : TVFDTPITLSHNQIRKLESTIMDLDNKTLWNDYRIAQPLNDRMVESSFLPRLGK--GTFCRQDEIESKILKIEGLITSLG : 312
ref|XP_013875818.1| : TVFDTPITLSHNQIRKLESTIMDIDNKTLSNDYRIPQPLNDRVVESSFLPRLGK--GTFCRQDEIESKILKIEGLITSLG : 313
ref|XP_005478239.2| : TVFDTPITLSHNQIRKLESTIMDMDNKTLWNDYRITQPLHERIVESSFLPRLGK--GTFCRQDEIESRILKIEGLITSIG : 312
ref|XP_006785611.1| : TVFDTPITLSHNQIRKLESTIMDMDNKTLWNDYRIAQPLNGRKVESSFLPRLGK--GTFCRQDEIESRIHKIEGLITSIG : 312
ref|XP_005914985.1| : TVFDTPITLSHNQIRKLESTIMDMDNKTLWNDYRIAQPLNGRIVESSFLPRLGK--GTFCRQDEMESRIHKIEGLITSIG : 312
ref|XP_004554282.1| : TVFDTPITLSHNQIRKLESTIMDMDNKTLWNDYRIAQPLNGRIVESSFLPRLGK--GTFCRQDEMESRIHKIEGLITSIG : 312
ref|XP_005733115.1| : TVFDTPITLSHNQIRKLESTIMDMDNKTLWNDYRIAQPLNGRIVESSFLPRLGK--GTFCRQDEMESRIHKIEGLITSIG : 312
ref|XP_010739228.1| : TVFDTPITLSHNQIRKLESTIMDMDNKTLWNDYRIAQPLNDRVVESSFLPRLGK--GTFCRQDEIESRIHKIEGLITSLG : 311
ref|XP_008317045.1| : TVFDTPITLSHNQIRKLESSIMDMDNKTLWNDYRMAQPLNDRVVESSFLPRLGK--GTFCRQDEIESKILKIEGLITSLG : 312
ref|XP_010793030.1| : TVFDTPITLSHNQIRKLEHTIMDIHNQTLWNDYRMAQPLNSRVVESSFLPRLGK--GTFCRQDEIESRILKIEGLITSLG : 313
ref|XP_008294025.1| : TVFDTPITLSHNQIRKLESTIMDMDNKTLWNDYRIAQPLNDRVVESSFLPRLGK--GTFCRQDEIESKILKIEGLITSLG : 312
ref|XP_002666525.1| : TVFDTPITLSHNQIRKLESTIMDHDNKTLWNDYRMAQPLNERVVESTFLPRLSK--GGMCRQEEIEAKIKRIESLILSLD : 310
ref|XP_009295179.1| : TVFDTPITLSHNQIRKLESTIMDHDNKTLWNDYRMAQPLNERVVESTFLPRLSK--GGMCRQEEIEAKIKRIESLILSLD : 310
ref|XP_007246352.1| : TVFDTPITLSHNQIRKLESTIMDLENKTLWNDYRMAQPLNDRVVESSFIPRLGK--GTFCRQDEIEAKISNIESMITSLG : 308
gb|KPP63671.1|      : TVFDTPITLSHNQIRKLEHTIMDLDNKTLWNDYRIAQPLNGRVVESTFRPRLGK--GTFCRQEEIEAKILKIEGLLTNLG : 276
ref|XP_012681508.1| : TVFDTPITLSHNQIRKLESTIMDHDNKTLWNDYRIAQPLNDRVVESSFLPRLGK--GSFCRQEEIEFKILKIESLITSLG : 279
ref|XP_010902628.1| : TVFDTPITLSHNQIRKLESTIMDMNNKTLWNDYRMAQPLNDRVVESSFLPRLGK--GTFCRQDEIETKILRIESLINSLG : 310
ref|XP_013989824.1| : TVFDTPITLSHNQIRKLESTIMDMANRTLWNDYRMAQPLNDRVVESSFLPGPRK--GAFCRHDEIESKILKIEGLITSLG : 310
ref|XP_014002952.1| : TVFDTPITLSHNQIRKLESTIMDMDNKTLWNDYRMAQPLNDRVVESSFLPRLGK--GTFCRQDEIESKILKIEGLITSLG : 310
ref|XP_007900354.1| : TVFDTPIKLSHNQIRLLENSVLDWENKTLRNDYRHAQLVNDRTIEASFQTDLVR---GSCHLDEINEKIEKIETILHKFQ : 294
ref|NP_001085550.1| : TVFDSPVFLSKTQIDLLENTILDWHNKTLRNDYRHAQPWHDRTVEASFHPKMPK---DSC-QQEISNKILLIETELQDMK : 311
ref|XP_005986136.1| : TVFDTPILLSYAQIQLLESTILDWKNKTLRNDYRHAQPLNDRVVKASFQPVFD----KGNYQEEINIKIEKIEGLVQDLK : 278
ref|XP_007432107.1| : TVFDSPIKLSYTQINLLENTILDWKNQTLRNDYRQIQPLNDRVVKASFSLQLVE---GRCRPESIASKINKIQTRLREMK : 306
ref|XP_006035521.2| : TVFDSPIILSPTQIKLLENTILDWQNKTLRNDYRHAQPLNDRVVESSFKAKVEQ---EICHLEELSLKIEQIQAQLQDVK : 308
ref|XP_014465858.1| : TVFDSPIILSPTQIKLLENTILDWQNKTLRNDYRHAQPLNDRVVESSFKAKVEQ---EICHLEELSLKIEQIQAQLQDMK : 308
ref|XP_005293083.1| : TVFDSPIILSHTQIKLLENTILDWQNNTLRNDYRHAQPLNDRVVESSFKAKGTK---ELCLPEELSSKIEQIQTQLQDLK : 308
ref|XP_006127158.1| : TVFDSPIILSHTQISLLENTILDWQNNTLRNDYRHTQPIHSRVVESSFKAKGTT---ERCLPEELNSKIEQIHTQIQEMK : 308
ref|XP_010213863.1| : TVFDAPVVLSHTQISLLESSILDWHNRTLRNDYRRAQPIHGRVVEASFRAPGK----ADTVTREKLKLRIEQIQMQLQDMK : 305
ref|XP_005057921.1| : SVFHSPIVLSHTQIQLLENTILDWHNRTLRNDYRHAQPIWGRVVEASFPAQRAQ---AQCHPEEFTLRIDQIQMQLQDLR : 307
ref|XP_002187446.1| : TIFHSPIVLSHTQVQLLENTILDWHNRTLRNDYRHAQPIRGRVVEASFRAPRGQ---AQCHPEEFTLRIDQIQMQLQDMK : 308
gb|KFO64797.1|      : TIFHSPVVLSHAQIRLLENSILDWHNRTLRNDYRHVQPIQGRVVEASFRAHRAQ---AQCHPEEFTLRIDQIQMQLQDMK : 283
ref|XP_425745.4|    : TIFDSPIVLSHTQIGLLENTILNWHNRTLRNDYRHAQPIHGRVVEASFQARPKQ---EQCQPEELSHKIEQIQTQLQDMK : 306
ref|XP_010721064.1| : TVFDSPIILSHAQIGLLENTILDWHNRTLRNDYRHAQPIHGRVVEASFQARPRQ---EQCQPEELSLKIEQIQTQLRDMK : 306
gb|KFW85044.1|      : TIFHSPIVLSHTQISLLENTILDWENRTLRNDYRHAQPIHGRVVESSFRARPPQ---AQCHPEELTLRIDQIQTQLQDMK : 282
ref|XP_013049674.1| : TIFDSPIVLSHTQISLLENTILDWHNQTLRNDYRHAQPIHGRLVEASFRAKPTP---EQCQPEELSLRIEQIQTQLQDMK : 309
gb|KFQ22427.1|      : TVFHSPIVLSHTQLSLLESSILDWHNRTLRNDYRHAQPIHGRVVQSSFRARLTP---EQCYPEEFNIRIEQIQEQLQDMK : 282
ref|XP_005143337.1| : TIFHSPILLSHTQISLLENTILDWQNRTLRNDYRHAQPIHGRVVEASFRSKPTQ---EQCHPEEFNLRIEQIQMQLQEMK : 308
gb|KFQ51874.1|      : TIFHSPILLSHTQISLLENTILDWQNRTLRNDYRHTQPIHGRVVEASFRAKATQ---EQCHPEEFDLRIEQIQMQLQDMK : 282
gb|KFP87788.1|      : TIFHSPIVLSHTQISLLENTILDWQNRTLRNDYRHAQPISGRVVESSFPARLNQ---AQCHPEEFTFRIDQIQMQLQEMK : 282
gb|KFP08430.1|      : TIFHSPIVLSHTQISLESTILDWQNRTLRNDYRHAQPLNHRMVESSFQAKLSQ---EQCSEEFSIRIEQIQMQLQEMK : 282
gb|KFP31739.1|      : TIFHSPIVLSHTQISLLENTILDWENRTLRNDYRHAQPIHGRVVEASFRARLTP---EQCHPEEINLRIEQIQMQLQDMK : 282
gb|EMC79327.1|      : TIFHSPIVLSHTQ--LLENTILDWQNRTLRNDYRHAQPLNGRAVESSFPAKPSP---EQCHPEEFGLRIEQIQMQLQDVK : 306
ref|XP_005237901.1| : TIFHSPIVLSHTQISLLENSILDWQNKMLRNDYRHAQPLNGRVVESSFRAELIH---EQCRPEEFSLRIEQIQVQLQDMK : 308
gb|KFV72304.1|      : TIFHSPIVLSHTQISLLEGTILDWHNRTLRNDYRHAQPLNGRVVESSFRPKPTQ---EQCHPEEFDLKIEQIQVQLQDMK : 282
gb|KFU95365.1|      : TIFHSPIVLSHTQISLLENTILDGQNRTLRNDYRHAQPLNGRVVESSFQAKPTP---EQCYPEEFSIRIEQIQMQLQDMK : 282
gb|KFU99210.1|      : TIFRSPIVLSHTQISLLENTILDWHNRTLRNDYRHAQPLNGRVVESSFRAQLTQ---EQCHPEEFNLRIEQIQMQLQDMK : 282
ref|XP_010076305.1| : TIFRSPIVLSHTQISLLENTILDWHNRTLRNDYRHAQPLNGRVVESSFRAQLTQ---EQCHPEEFNLRIEQIQMQLQDMK : 307
gb|KFQ39105.1|      : TIFHSPIVLSHTQIRLLENTILDWHNRTLRNDYRHAQPLNGRVVESSFRAQLTQ---EQCHPEEFTLRIEQIQMQLQDVK : 288
gb|KFW07663.1|      : TIFHSPIVLSHTQISLLENTILDWHNRTLRNDYRHAQPLNGRVVESSFRAKLTQ---EQCPPEDFSLRIEQIQRQLQDMK : 281
gb|KFO73096.1|      : TIFHSPIVLSHTQINLLENTILDWQNRTLRNDYRHAQPLNGRVVESSFRAKRTQ---EQCHPEEFSLRIEQIQTQLQDMK : 282
gb|KFV05980.1|      : TIFHVPIVLSHTQISLLENTILDWHNRTLRNDYRHAQLLNGRVVESSFRAKLTQ---EQCHPEEFNLRIEQIQMQLQDMK : 282
gb|KFO89464.1|      : TIFHSPIVLSHTQISLLEGTILDWQNRTLRNDYRHAQPLNGRVVESSFRAKRTQ---EQCHPEELSLRIEQIQVQLQDVK : 291
gb|KFP34798.1|      : TIFHSPIVLSHTQISLLENTILDWQNRTLRNDYRHAQPLNGRVVESSFRAKLTQ---EQCHPEEFNLRIEQIQLQLQDMK : 282
gb|KFW93611.1|      : TIFNSPIVLSHTQISLLENTILDWQNRTLRNDYRHAQPLNGRVVESSFRAKPAQ---EQCHPEEVNLRIEQIQMQLQDMK : 282
gb|KFP22238.1|      : TIFHSPIVLSHTQISLLENTILDWQNRTLRNDYRHAQPLNGRVVESSFRAKLTQ---EQCRPEEFDLRIEQIQMQLQDMK : 282
gb|KFQ78929.1|      : TIFHSPIVLSHTQISLLENTILDWQNRTLRNDYRHAQPLNGRVVESSFRATLTQ---ERCHPEEINLRIEQIQMQLQDMK : 282
gb|KFP87033.1|      : TIFHSPIVLSHTQISLLENTILDWQNRTLRNDYRHAQPLNGRVVESSFRAKLTQ---EQCHPEEVSLRIEQIQVQLQDMR : 281
gb|KGL96941.1|      : TIFHSPIVLSHTQISLLENTILDEQNRTLRNDYRHAQPLNGRVVESSFRAKFTQ---EQCHPEEFHLRIEEIQVQLQDMK : 282
gb|KFZ60550.1|      : TIFHSPIILSHTQISLLESTILDWHNRTLRNDYRHAQPLNGRVVESSFRAKLTQ---EQCQPEEFNLRIEQIQMQLQDMK : 282
gb|KFP94842.1|      : TIFHSPIVLSHTQISLLENTILDWENRTLRNDYRHAQPLNGRVVESSFRAKLTQ---EQCHAEEFNLRIEQIQMQLQDMK : 282
ref|XP_011583597.1| : TIFHSPIVLSHTQISLLENTILDWQNRTLRNDYRHAQPLNGRVVESSFKAKLTQ---EQCHAEEFNLRIEQIQMQLQDMK : 308
gb|KFZ62512.1|      : TIFHSPIVLSHTQISLLENTILDWQNRTLRNDYRHAQPLNGRVVESSFSTKLAQ---ERCHPEEFSLRIEQIQMQLQDVK : 282
gb|KFQ06064.1|      : TIFHSPVVLSHIQISLLENTILDWQNKTLRNDYRHAQPLNGRVVESSFRAQLTQ---EQCQPEEFSLRIEQIQMQLQDMK : 282
ref|XP_009965819.1| : TIFHSPIVLSHTQVSLLENTILDWQNRTLRNDYRHAQPLNGRVVESSFRARLTQ---EQCHPEEFNLKIEQIQMQLLDMK : 308
gb|KFV47237.1|      : TIFQSPIVLSHTQISLLENTILDWQNKTLRNDYRHAQPLNGRVVESSFRAKLTQ---EPCRSEVVNLRIEQIQMQLRDMK : 282
gb|KFO11499.1|      : TIFHSPIILSHTQISLLENTILDWQNRTLRNDYRHAQPLNGRVVESSFRAKRTQ---EQCHTEEFNLRIEQIQMQLQDMK : 282
gb|KFQ78854.1|      : TIFHSPIVLSHTQISLLENTILDWQNRTLRNDYRRAQPLNGRVVESSFRAKLTQ---EQCYPEEFNLRIEQIQMQLQDMK : 282
gb|KFW11285.1|      : TIFHSPIVLSHTQISLLENTILDWQNKTLRNDYRHAQPLNGRVVESSFRAKLIQ---EQCHPEEFNLRIEQIQMQLQDMK : 282
gb|KFQ66673.1|      : TIFHSPIVLSHTQISLLENTILDWQNRTLRNDYRHAQPLNGRVVESSFRAKLTQ---EQCHPEEFNLRIDQIQMQLQDMK : 282
gb|KFQ93800.1|      : TIFHSPIVLSHTQISLLENTILDWQNRTLRNDYRHAQPLNGRVVESSFRAKLTQ---EQCDPEEFTLRIEQIQMQLQDMK : 282
gb|KFP53434.1|      : TIFHSPIVLSHTQISLLENTILDWQNRTLRNDYRHAQPLNGRVVESSFRAKLTQ---EQCHPEEFSLRIEQIQMQLQDMK : 282
ref|XP_009323546.1| : TIFHSPIVLSHTQISLLENTILDWQNRTLRNDYRHAQPLNGRVVESSFRAKLTQGKGKQCHPEEFNLRIEQIQMQLQDVK : 311
gb|KFM01971.1|      : TIFHSPIVLSHTQISLLENTILDWQNRTLRNDYRHAQPLNGRVVESSFRAKLTQ---EQCHPEEFNLRIEQIQMQLQDMK : 282
                      t F  Pi LSh Qi  LE tl d  N tL NDYR aQpln RvvessF         c  e   l I
```

```
                                    *       340         *       360         *       380         *       400
ref|XP_012726090.1| : DMYLGGH---PRSRHSK---------------YDHSDLFPLVLNFEDRNTQSYALAHLSHLIDLDAFTVCMHVLLK-AGG : 368
ref|XP_007570993.1| : KNMYSGIGGRSSSRHSK---------------YDHGELFPLVLNFEDRNSVSYALAPL-SRMTLDAFTVCMHVLLR-APG : 374
ref|XP_008411549.1| : KNMYSGIGGRPSSRPSK---------------YEQGELFPLVLNFEDRNSVSYALAPL-GRMDLDAFTVCMHVLLR-APG : 374
ref|XP_004070439.1| : KNLYSG-GNRS-PNPPR---------------YDHSDLFPLVLGFPERNYGSYALARLNHPMDLDSFTVCMHVLPQ-GPG : 371
emb|CAF93821.1|     : KQLYA----------------------------ERTGLFPLVLNFQHSNDITYALAHVSHP-DMNAFTVCLHVLPQ-DQG : 361
ref|XP_011620264.1| : KYLYAG-GIRGSGPQNK---------------NEHTGLFPLVLNFQDNNDGGYALAHVSHPIYLNAFTICMHVLSK-PEE : 375
ref|XP_013875818.1| : KNMYSG-DV-PGSRPSR---------------YDRGGLFPLVLSFNDKNAISYALTHLTHPMDLDSFTVCVHVLLK-VEG : 375
ref|XP_005478239.2| : KNIHSG-GFHN-SRPTR---------------YETGSLFPLVLNFQEKDSDSYALASLSHPMDLDAFTVCMHVLPQ-IEG : 374
ref|XP_006785611.1| : KNIHSG-GFHN-SRPTR---------------HETGSLFPLVLNFREKDSGSYTLASLSHPMDLDAFTVCMHVLPQ-VEG : 374
ref|XP_005914985.1| : KNIHSG-GFHS-SRPTR---------------HETGSLFPLVLNFREKDSGSYALASLSHPMDLDAFTVCMHVLPQ-VEG : 374
ref|XP_004554282.1| : KNIHSG-GFHS-SRPTR---------------HETVSLFPLVLNFREKDSGSYALASLSHPMDLDAFTVCMHVLPQ-VEG : 374
ref|XP_005733115.1| : KNIHSG-GFHS-SRPTR---------------HETGSLFPLVLNFREKDSGSYALASLSHSMDLDAFTVCMHVLPQ-VEG : 374
ref|XP_010739228.1| : KHMHSG-GFRN-PRPAT---------------YESRGLFPLVLNFQEQNAGSYALARLSHPMDLSAFTVCMHVLPQ-LKG : 373
ref|XP_008317045.1| : RIVHSG-GQRN-TKASR---------------NEPRALFPLVFNFPEKNSGSYALARLSHSMDLDSFTVCMHVLPQ-IEG : 374
ref|XP_010793030.1| : KHIHTS-GHHNPSRPTR---------------LETRALFPLVLHFQEKNSGSYALARLGHPMELDSFTVCMHVRPQ-LEG : 376
ref|XP_008294025.1| : KNMHSG-GLRN-PRPTR---------------YEPRALFPLVLNFQEKNAGSYALARLSHPMDLDSFTVCMHVLPQ-VEG : 374
ref|XP_002666525.1| : KKAVQG-----------------------------KQPISPLVLYFPQKNVESFAVVNLTHPMELKSFTACMNVQIP-PIR : 361
ref|XP_009295179.1| : KKAVQA-----SRPSST---------------GGKQPISPLVLYFPQKNVESFAVVNLTHPMELKSFTACMNVQIP-PIR : 369
ref|XP_007246352.1| : KHVYSEA--SGSKP--R---------------DKDEAIYPLVLHFPENKLGSYAVVNVAHSMNLHSFTACMNLYMR-PRP : 368
gb|KPP63671.1|      : KQVNSG----------------------------ARVITPLVLQFTERRTESYALVHSTHTMQLQAFTACMHVRIV-PGG : 327
ref|XP_012681508.1| : KYIRTSA--MGDARPTR-----------------PRALYPLVLHFTERNTGSYALAHLTHPMSLQSFTACMGIRTRYPGE : 340
ref|XP_010902628.1| : KQMHSV----------------------------SRVLSPMVLHFPERNTRSYAMAHLTHPMDLHSFTACMHVKTP-PVG : 361
ref|XP_013989824.1| : KHIRSSA--IGDARPSE---------------HEPRAMSPLVLHFPESNTESYARAHLAHSMDLHSFTACMHLRTR-PGW : 372
ref|XP_014002952.1| : KHIHSSA--IGDARPSK---------------QEPRVMSPLVLHFPERNTESYARAHLAHSMDLHSFTACMHLRTR-PGE : 372
ref|XP_007900354.1| : LNAEAGQA------------------------GIELASHP-AFHFSEGRRESYVEIRTLRSMDLRSFSVCMWIRTK-DSR : 348
ref|NP_001085550.1| : KRADSSKSRL----------------------LVSGSPSYP-SFYFSKDHLAARVEVRPAESMKMARFTICVWVRTK-NEG : 368
ref|XP_005986136.1| : HMLPNAT-------------------------GNQHSSFP-AFKFLQETTLSFVEVTPLWTMELNSLTSCMWISTQ-NEG : 331
ref|XP_007432107.1| : KYLLDVIGKP----------------------GHHQRSLQ-AFHFPLENTASYVTVIPLKPMQLKTFTLCFWVQNR-NQG : 362
ref|XP_006035521.2| : KHLLGGVHLR----------------------GAQSSAFP-AFFFPADNIESYVNVRPRHVMTLHSFTLCFWIQTQ-NRG : 364
ref|XP_014465858.1| : KHLLGGVHLR----------------------GAQSSAFP-AFFFPADNIESYVNVRPRHVMRLHSFTLCFWIQTQ-NLG : 364
ref|XP_005293083.1| : KHLLAGVGLT----------------------GLKSSAFP-AFYFPAENIASYVAVRPLRDMKLQAFTLCFWIQAQ-NRG : 364
ref|XP_006127158.1| : KHLLAGGGMT----------------------SHKSSAFP-AFYFPAENIISYVTVRPLRSMQLLSFTLCFWIHAQ-NQG : 364
ref|XP_010213863.1| : KELLDGVSQI----------------------GVKSSPFP-AFYFPAENIESSVTVQPLHDMALQAFTLCFWARAA-AGG : 361
ref|XP_005057921.1| : TELLNGLSHT----------------------DTKCSTFP-AFYFPVENIESFVEVHPLRAVSLQAFTLCFWSRAQ-PVG : 363
ref|XP_002187446.1| : TELLNGLSHT----------------------GTKSSTFP-AFYFPVENIESFVEVHPLRAVSLQAFTLCFWSRAQ-PVG : 364
gb|KFO64797.1|      : TELLNGLSHT----------------------GTKSSTFP-AFYFPVENIESFVEVHPLRAVSLQAFTLCFWSRAQ-PAG : 339
ref|XP_425745.4|    : KEFLNGVSHT----------------------GITCSTFP-AFYFPMENIESFVEVRPRHEMSLQAFTLCFWTQAQ-HVG : 362
ref|XP_010721064.1| : KEFLNGVSHT----------------------GITSSTFP-AFYFPMENIESFVEVRPSHEMSLQAFTLCFWTQAQ-HVG : 362
gb|KFW85044.1|      : TELLNRGSQA----------------------GVKPSAFP-AFYFPVENIESFVDVHPLRDMSLQAFTLCFWARSR-HPG : 338
ref|XP_013049674.1| : KELLNGVSHVGKKNVYSQVQLTFQTAGFLVISGIKSSSFP-AFYFPVENIESFVTVHPLHDMSLQAFTLCFWTKAQ-HAG : 387
gb|KFQ22427.1|      : RELLSGVSHT----------------------GIKSSTFP-AFYFPVENIESLVSIDPLRDMSLQAFTFCFWAKAQ-HAG : 338
ref|XP_005143337.1| : RELLNGGSHI--D----------VTAGFLVVSGIKSSTFP-SFYFPVENIESFVNIHPLHDMSLQAFTLCFWIKAQ-HAG : 374
gb|KFQ51874.1|      : RELLNGVSHV----------------------GIKSSTFP-SFYFPVENIESFVNVHPLHDMSLQAFTLCFWTKAQ-HAG : 338
gb|KFP87788.1|      : TELLNGVSHT----------------------GLKSSTFP-AFYFPAENIESFVNVHPLQDTSLQAFTLCFWTKAQ-HSG : 338
gb|KFP08430.1|      : RELLTGVSHI----------------------GIKSCTFP-AFYFPVENFESFVNVHPLHDMSLQAFTLCFWTKAQ-RAG : 338
gb|KFP31739.1|      : TELLTGLSPM----------------------GIKSSTFP-AFYFPVENIESFVNVHPLHDMSLQAFTLCFWTKAH-HAG : 338
gb|EMC79327.1|      : SELLKGMSHI----------------------GIKSSTFP-AFYFPAENIESFVDIHPLHDMSLQAFTLCFWSKAQ-PTG : 362
ref|XP_005237901.1| : REFLNGVNYI----------------------GIKSSTFP-AFYFPMENIESSVNVHPLHDMSLQGFTLCFWTKAQ-HAG : 364
gb|KFV72304.1|      : RELLNGVSHT----------------------GLKSSTFP-AFYFPVENIESFVTVHPLHDMSLQAFTLCFWTKAQ-HDG : 338
gb|KFU95365.1|      : RELLNGVSHI----------------------GMTSSTFP-AFYFPVENIESFVNVHPLHGMSLQAFTLCFWTKAQ-SVG : 338
gb|KFU99210.1|      : RELLNGVSHV----------------------GTKSHTFP-AFYFPVENIESFVNVHPLHDMSLQAFTLCFWTKAQ-HAG : 338
ref|XP_010076305.1| : RELLNGVSHVAGN----------VTAAFLVISGTKSHTFP-AFYFPVENIESFVNVHPLHDMSLQAFTLCFWTKAQ-HAG : 375
gb|KFQ39105.1|      : RELLNGVSHI----------------------GIKSSTFP-AFYFPVENIESLVSVHPLHEMSLQAFTLCFWTKAQ-HAG : 344
gb|KFW07663.1|      : REMLNGVSHV----------------------GTKSSTFP-AFYFPVENIESFVNVHPLHDMSLQAFTLCFWTKAQ-HAG : 337
gb|KFO73096.1|      : SELLNRASHI----------------------GIKSSTFP-AFYFPVENIESFVKVHVLHDMSLQAFTLCFWTKAQ-HAG : 338
gb|KFV05980.1|      : KDLLNGVSHV----------------------GIKSSTFP-AFYFPVENIESFVTVQPLRDMSLQAFTLCFWAKAQ-HAG : 338
gb|KFO89464.1|      : RELLNGVSHV----------------------GVQSSTFP-AFYFPVENIESFVNVHPLHAMSLQAFTLCFWTKAQ-HAG : 347
gb|KFP34798.1|      : KELLNGVSHI----------------------DIQSSTFP-AFYFPAENIESFVDVHPLHDMSLQAFTLCFWAKAQ-RAG : 338
gb|KFW93611.1|      : RELLNGVSHI----------------------GIKSSTFP-AFYFPVENIESFVNVHPLHDISLQAFTFCFWAKTQ-HAG : 338
gb|KFP22238.1|      : RELLNGVSHV----------------------GIKSSAFP-AFYFPVENIESFVTVQPLHEMSLQAFTLCFWTKAQ-HAG : 338
gb|KFQ78929.1|      : RELLNGVRHI----------------------GIKSSTFP-AFYFPVENIESFVNVHPLHDMSLQAFTLCFWTKAQ-RAG : 338
gb|KFP87033.1|      : RELLNGLRHT----------------------GIQSSTFP-AFYFPVENIESFVNVHPLHDMSLQAFTLCFWTKAQ-HAG : 337
gb|KGL96941.1|      : RELLNGVSHI----------------------GIKSSTFP-AFYFPVENIESFVTVRPLRDMSLQAFTLCFWTKAQ-HAG : 338
gb|KFZ60550.1|      : RELLNGVSHI----------------------GIKSSTFP-AFYFPVENIESFVNVHPLHDMSLQAFTLCSWTKAQ-HTG : 338
gb|KFP94842.1|      : RELLNGVSHI----------------------GIKSSTFP-AFYFPVENIESFVNVHPLHDMSLQAFTLCFWTKAQ-HAG : 338
ref|XP_011583597.1| : RELLNGVSHI----------------------GIKSSTFP-AFYFPVENIESFVNVHPLHDMSFQAFTLCFWTKAQ-HAG : 364
gb|KFZ62512.1|      : KELLNGASHI----------------------GIKSSAFP-AFYFPVENIESFVNVHPLQDMSLQAFTLCFWTKAQ-HAG : 338
gb|KFQ06064.1|      : REFLNGASHI----------------------GIKSSTFP-AFYFPVENIESFVNVHPLHDMSLQAFTLCFWTKAQ-PAG : 338
ref|XP_009965819.1| : SKLLNGVSHIAGN----------VTAGFWLISGIKSSTFP-AFYFPVENIESFVYVHPLRDMSLQAFTLCFWTKAQ-HAG : 376
gb|KFV47237.1|      : RELLNGVSHI----------------------GIKSSTFP-AFYFPVENIESFVNVHPLHDMSLQAFTLCFWTKAQ-HSG : 338
gb|KFO11499.1|      : RELLNGVSHT----------------------GIRSSTFP-AFYFPVENIESIVNVRPLHDMSLQAFTLCFWTKSQ-HAG : 338
gb|KFQ78854.1|      : RELLNGVSHI----------------------GIKSSTFP-AFYFPVENIESFVNVHPRHDMSLQAFTLCFWTKAQ-HAG : 338
gb|KFW11285.1|      : RELLNGVSHI----------------------GIKSNTFP-AFYFPVENIESFVNVHPLHDMSLQAFTLCFWARAQ-RAG : 338
gb|KFQ66673.1|      : RELLNGVSHF----------------------GIKSSTYP-AFYFPVENIESFVNVHPLHDMSLQAFTLCFWTKAQ-HAG : 338
gb|KFQ93800.1|      : KELLNGVSHI----------------------GIKSSTFP-AFYFPVENIESFVNVHPLHDMSLQAFTLCFWTKAQ-HAG : 338
gb|KFP53434.1|      : RELLNGVSHI----------------------GIKSSTFP-AFYFPVENIESFVKVHPLHDMSLQAFTLCFWTKAQ-HAG : 338
ref|XP_009323546.1| : RELLNGVSHI----------------------GIKSSTFP-AFYFPVENIESFVNVRPLHDMSLQAFTLCFWTKAQ-HAG : 367
gb|KFM01971.1|      : RELLNGVSHI----------------------GIKSSTFP-AFYFPVENIESFVNVHPLHDMSLQAFTLCFWTKAQ-HAG : 338
                           g                       fp   F  n s       m l  ft C       g
```

```
                                  *        420         *        440         *        460         *        480
ref|XP_012726090.1| : VHTVISYSSGGHDNELMVSISQDTSAMEVGLWIGNEFVNMPHNTKTQDWANYCITWSSHSGGVDLWINGMVGESRHLQSS : 448
ref|XP_007570993.1| : VHTVISYSSSIHDNELMISISQDANAREVGLWIGDDFVNMPHGFNTQDWTNYCVTWSSHSGGVDLWINGKVGEQRHLQSS : 454
ref|XP_008411549.1| : VHTVLSYSSGIHDNELMVSISEDANAREVGLWIGDDFVNMPHGFSTQDWTNYCVTWSSHSGGVDLWINGKVGEQRHLQSS : 454
ref|XP_004070439.1| : VHTVISYSSDGNDNELTVSISKDAESGEIGLWIGNEFVNMPHTFRSQDWTNYCITWSSHTGGADIWVNGMLGEQRHLRSG : 451
emb|CAF93821.1|     : SQTVLSYTSSEHENEVAISISADGDSREVGLWIGNEFVNLPHSFKAHVWTNYCVTWSSQTGGAELWINGMVGEQRHLKRG : 441
ref|XP_011620264.1| : INTVLSYSSSEHDNEITISITEDMNAREVGLWIGNAFVNLPHSFRAHDWTNYCVTWSSDLGAAELWINGMVGEQRYLKSG : 455
ref|XP_013875818.1| : SHTVLSYSSHGVDNELMISINEDSDAREVGLWIGNEFINLPHNFMSQDWTNYCVTWSSHTGGAELWVNGKVGEQRYLRSS : 455
ref|XP_005478239.2| : VHTVISYSSSGNDNELMISLGEDRDAREVGLWIGNEFVNLPHNFKPKDWANYCVTWSSHTGGAELWINGRVGEQRYLRSG : 454
ref|XP_006785611.1| : VHTVISYSSNGNDNELMISLGEDRDAREVGLWIGNEFVNLPHNFKPKDWVNYCITWSSHTGGAELWINGMVGEQRYLRRD : 454
ref|XP_005914985.1| : VHTVISYSSNGNDNELMISIGEDRDAREVGLWIGNEFVNLPHNFKPKDWVNYCITWSSHTGGAELWINGMVGEQRYLRRD : 454
ref|XP_004554282.1| : VHTVISYSSNGNDNELMISLEEGRDAREVGLWIGNEFVNLPHNFKPKDWVNYCITWSSHTGGAELWINGMVGEQRYLRRD : 454
ref|XP_005733115.1| : VHTVISYSSNGNDNELMISLEEGRDAREVGLWIGNEFVNLPHNFKPKDWVNYCITWSSHTGGAELWINGMVGEQRYLRRD : 454
ref|XP_010739228.1| : LHTAISYASNGNDNELMISISEDRDAKEVGLWIDNEFINLPHNFRSHDWANYCITWSSNNGGVQLYINGMVGEQQHLKSG : 453
ref|XP_008317045.1| : IHTVISYASNSNENELMISIGEDRDGKEVGLWIGNEFVNLPHNFKSRDWANYCVTWSSHTGGAELWINGMVGEQRYLQSG : 454
ref|XP_010793030.1| : VHTVISYSSNGNDNELTISIVEDRDSREVGLWIGNEFVNLPHNFKSHKWANYCITWSSLTGGAELWINGMVGEQRYLKNG : 456
ref|XP_008294025.1| : IHTVISYSSNGNDNELMISIGEDRDSKEVGLWIGNEFVNLPHNFKSHDWANYCITWSSHTGGAELWINGMVGEQRYLKSG : 454
ref|XP_002666525.1| : DLTVLSYST-SHDNELMISLG-----SEVGLWIGDEFVNLSFDLPSSDWTNYCLTWASHNGGAELWVNGVVGKERYIRTG : 435
ref|XP_009295179.1| : DLTVLSYST-SHDNELMISLG-----SEVGLWIGDEFVNLSFDLPSSDWTNYCLTWASHNGGAELWVNGVVGKERYIRTG : 443
ref|XP_007246352.1| : VHTVLSYSSSGNDNELTITMG-----HEIGLWIGNEFVNLPHNHAHSWAHYCVTWASHTGGAEVWINGVVGDEQYLRAG : 443
gb|KPP63671.1|      : VHTVLSYSTSTNDNELVISVG-----YEVGLWIGNAFLNFPLDLQSTEWVNYCLTWASHSGGVELSVNGMVGEEQYLQSG : 402
ref|XP_012681508.1| : AHTIFSYSTPAHDNELVVTMG-----QDVGLWIGNEFVSLPHDLRAFNWAHYCMTWASHTGGAELWINGMVGEEQYLRAG : 415
ref|XP_010902628.1| : IHTVLSYSSQGKDNELMISIG-----YEVGLWIGNEFVNLPHNFHSHEWANYCVTWSSHSGGADLTINGLVGEEQYIRGG : 436
ref|XP_013989824.1| : VHTVLSYYSQGNDNELMITMG-----YEVGLWIGNEFVNLPHNFHSRDWANYCVTWSSHSGGAELWINGVVGEEQYLQRG : 447
ref|XP_014002952.1| : IHTVLSYSSQGNDNELMITMG-----YEVGLWIGNEFVNLPHNFHSRDWVNYCVTWSSHSGGVELWINGLVGEEQYLRRG : 447
ref|XP_007900354.1| : SKILLSYATKGSDNELVISVG-----LDWGLWIGGHFVNIGFHFNSKDWVHYCLTWRSQSGSAEMWINGLSGKEKRIKRG : 423
ref|NP_001085550.1| : SQTIFSYSTNNRDNELVLSVG-----NDIGVWIGGEFINFNIHHTSEDWVHYCVSWDSQNGAAKLWVSGLPGKEKTIQHG : 443
ref|XP_005986136.1| : TKAVFSYAMFETDNELVITVG-----SDVGLWIGGYFVSFPLHHRTEDWVYYCVTWTSNTGLAELWINGIVGKGKLIKKG : 406
ref|XP_007432107.1| : RQTVLFYSTPESENELVISVG-----MAVGVWIGGKFVQFDLHRRAEDWVHHCVTWLSSSGTINLWVNGAVGTARNIQKN : 437
ref|XP_006035521.2| : SQTVLSYSTPESDNELVVTVG-----SDVGLWVGGHFINFFLHHRSEEWLHYCVSWTSHSGTADLWINGAVGREKSIQKG : 439
ref|XP_014465858.1| : SQTVLSYSTPESDNELVVTVG-----SDVGLWVGGHFTNFFLHHRSEEWLHYCVSWTSHSGTADLWINGAVGREKSIQKG : 439
ref|XP_005293083.1| : SQTILAYCTQERDNELVVTVG-----SAVGLWVGGHFVEFPLHHKSEEWLHYCVAWASHSGAANLWLNGAAGREKHLQRG : 439
ref|XP_006127158.1| : SQTVLSYSTQERDNELLVTVG-----SAVELWVGGYSVEFPLHHTSEEWLHYCVTWASHSGAAHLWINGAVGREKHLQRG : 439
ref|XP_010213863.1| : TQTVLSYSTQQRDAELVVTVG-----AAVALWVGGRVASFPTHHGSQGWLHHCVAWASPTGRAQLWLNGAEGKAQSVQKG : 436
ref|XP_005057921.1| : SQTVLSYSTGESDHELVVTVG-----TELGLWVGGHFLSFPLQQEARGWLHHCVTWCSQEGTANLWLNGAAGKAQSLQQG : 438
ref|XP_002187446.1| : SQTVLSYSTGERDRELAVTVG-----SELGLWVGGHFLSFPLQQEAQGWLHHCVTWASPEGRASLWLNGAAGQAQSVQKG : 439
gb|KFO64797.1|      : SQTILSYSTGESDHELLVTVG-----AELGLWIGGHFLSFPLQHGAQDWLHHCVTWASQAGAANLWLNGAAGKAQSVQKG : 414
ref|XP_425745.4|    : TQTVLSYSTRERDNELVMTTG-----TEVGLWIGGHFVSFPLYHKARDWLHYCMAWASQSGIANLWLNGAAGDAKSIQRG : 437
ref|XP_010721064.1| : TQTVLSYSTRERDNELVMTTG-----TDVGLWIGGHFVSFPLYRKAQDWLHYCMAWASQSGIASLWLNGAAGDAKSIQKG : 437
gb|KFW85044.1|      : SQTVLSYSTRERDNELLVSVG-----TEVGLWVGGHSISFPLHHKAQDWLHYCLAWASQPGTANLWLNGAAGKAASVQKG : 413
ref|XP_013049674.1| : SQTILSYSTQERDNELVLTVG-----TDVGLWIGGHFISFPLYHKAQDWLHFCMTWASQSGMANLWLNGAAGKAKSIQKG : 462
gb|KFQ22427.1|      : TQTVLSYSTQERDNELVMTVG-----TDAGLWLGGHFISFPLYHKAQDWLHYCLVWDSQSGMANLWLNGAAGKAKSIQKG : 413
ref|XP_005143337.1| : SQTVLSYSTQERDNELVLTVG-----TDVGLWIGGHFISFPLYHKAQDWLHYCMTWDSWSGMANLWLNGAAGKAKSIQKG : 449
gb|KFQ51874.1|      : SQTVLSYSTQERDNELVLTVG-----TDVGLWIGGHFISFPLYHKAQDWLHYCVAWASWSGTASLWLNGAAGTAKSIQKG : 413
gb|KFP87788.1|      : SQTILSYSTQERDNELVVTVG-----TEVALGIGDHFISFPLSHKAQDWLHYCVAWASQSGMANLWVNGAAGKEMSIQKG : 413
gb|KFP08430.1|      : SQTVLSYSTQERDNELVMTVG-----TAVGLWIGGHFISFPLYNMAQDWLHYCMAWSSQSGMANLWLNGAPGKAKSIQKG : 413
gb|KFP31739.1|      : SQTVLSYSTQERNNELAMTVG-----TDVGLWIGGHFTSFPLSHKAEGWLHYCLAWASQSGMANLWLNGAVGKAKSIQKG : 413
gb|EMC79327.1|      : SQTVLSYSTQERDNELVVTVG-----AEVGLWIGGHFISFPLYHRAQVWLHYCMAWASQSGMANLWLNGAAGEAKSIQKG : 437
ref|XP_005237901.1| : SQTVISYSTQERDNELVVTVG-----TDVGLWIGGHFISFPLYHKAQDWLHYCLAWASQSGMANLWLNGAAGKAKSIQKG : 439
gb|KFV72304.1|      : TQTVLSYSTQERDNELVMTVG-----SDVGLWIGGHFISFPLHHKAQDWLHYCVAWASQSGMANLWLNGAAGKEKSIQQG : 413
gb|KFU95365.1|      : SQTVLSYSTQERDNELVLTVG-----SDVGLWIGGHFISFPLYHMAQGWLHYCMAWASQSGMANLWLNGAAGKAKSIQKG : 413
gb|KFU99210.1|      : SQTVLSYATQERDNELVVTVG-----TEVGLWIGGHFISFPLYHKAKDWLHYCMAWASQAGMANLWLNGAAGKAKSIQKG : 413
ref|XP_010076305.1| : SQTVLSYATQERDNELVVTVG-----TEVGLWIGGHFISFPLYHKAKDWLHYCMAWASQAGMANLWLNGAAGKAKSIQKG : 450
gb|KFQ39105.1|      : TQTVLSYSTQERDKELVMTVG-----TDVGLWIGGHFISLPLYREAEDWLHYCVAWASQSGMANLWLNGAAGKAKSIQKG : 419
gb|KFW07663.1|      : SQTVLSYSTQERDNELVMTVG-----TDAGLWIGGHFISFPLYHKAQDWLHYCMAWASQSGMANLWLNGAAGKAKSIQKG : 412
gb|KFO73096.1|      : SQTVLSYSTQEREKELVMTVG-----TEVGLWIGSHFISFPLNHKAQDWLHYCMAWASQSGTANLWLNGAAGKEKSIQKG : 413
gb|KFV05980.1|      : SQTVLSYSTQERDNELVVTVG-----TDVGLWIGGHSISFPLYYKAQDWLHYCMAWASHSGMANLWLNGAAGKAESIQKG : 413
gb|KFO89464.1|      : SQTVLSYSTQERDKELVMTMG-----TDVGLWLGGHFISFPLYHKAQDWLHYCMEWASQSGMANLWLNGAAGKAKSIQKG : 422
gb|KFP34798.1|      : SQTVLSYSTQERDKELVMTVG-----TDVGLWIGDHFISFPLHHKAQDWSHYCVAWASQSGMANLWLNGAAGKGKSIQKG : 413
gb|KFW93611.1|      : SQTVLSYSTQERDNELVVTVG-----ADVGLWIGGHFISFPLYHKAQGWLHYCMAWASQSGMANLWLNGAAGKEKSIQKG : 413
gb|KFP22238.1|      : SQTVLSYSTQERDNELAVTVG-----TDVGLWIGGHFISFPLDHKAQDWLHYCMAWASQSGMANLWLNGAPGKAKSIQKG : 413
gb|KFQ78929.1|      : SQTVLSYSTQQRDNELVMTVG-----TDVGLWIGGHFISFPLYHNAQDWLHYCTAWASQSGMANLWLNGAAGKAKSIQKG : 413
gb|KFP87033.1|      : SQTVLSYSTQERDNELVMTVG-----TDVGLWIGGHFISFPLQHEAQDWLHYCMAWASQSGMANLWLNGAAGKAKSIQKG : 412
gb|KGL96941.1|      : SQTVLSYSTQERDNELVMTVG-----TDVGLWIGGHFISFPLYHRAQNWLHYCMAWASQSGMAILWLNGAAGKAKSIQKG : 413
gb|KFZ60550.1|      : SQTVLSYSTQERDNELVMTVG-----TDVGLWIGGHFISFPLYHKAQDWLHYCMAWASQSGMANLWLNGAAAKAVSIQKG : 413
gb|KFP94842.1|      : SQTVLSYSTQERDNEIVITVG-----TDVGLWIGGHFITFPLYHKEQDWLHYCMAWASESGIANLWLNGAAGKAKSIQKG : 413
ref|XP_011583597.1| : SQTVLSYSTQERDNEIVITVG-----TDVGLGIGGHFISFPLYHKEKDWLHYCMAWASESGMANLWLNGAAGKAKSIQKG : 439
gb|KFZ62512.1|      : SQTVLSYSTQERDNELVMTVG-----TDVGLWIGGQFISFPLYHKAQDWLHYCTTWASQSGMANLWLNGVAGKAKSIQKG : 413
gb|KFQ06064.1|      : SQTVLSYSTQERDNELVMTVG-----TDVGLWIGGHFISFPLYYKAQDWLHYCMAWASQSGMASLWLNGAAGKAKSIQKG : 413
ref|XP_009965819.1| : SQTVLSYSTQERDNELVMTVG-----TDVGLWIGGHCISFPLYHKAEDWLHYCMAWASQSGMANLWLNGAAGKGKSIQKG : 451
gb|KFV47237.1|      : SQTVLSYSTQERDNELVMTVG-----TDVGLWIGGHFISFPLYQKAQDWLHYCMAWASQSGMANLWLNGAAGKAKSIQKG : 413
gb|KFO11499.1|      : SQTVLSYSTQERDNELVMTVG-----TDVGLWIGGHFISFPLYHKAQDWLHYCMAWASQSGMANLWLNGAAGEAKSIQKG : 413
gb|KFQ78854.1|      : SQTVLSYSTQERDNELVVTVG-----TDVGLWIGGHFISFPLHHKAQDWLHYCVAWASQSGMANLWLNGAAGKAKSIQKG : 413
gb|KFW11285.1|      : SQTVLSYSTQERDNELVMTVG-----TDVGLWIGGHFISFPLYHKAQDWLHYCMAWASQTGMANLWFNGAAGKAKSIQKG : 413
gb|KFQ66673.1|      : SQTVLSYSTQERDNELVMTVG-----TDVGLWIGGHFISFPLYHKAQDWLHYCMAWASQSGMANLWLNGAAGKAKSIQKG : 413
gb|KFQ93800.1|      : SQTVLSYSTQERDNELVMTVG-----TDVGLWIGGHFISFPLYHEAQDWLHYCMAWASQSGMANLWLNGAAGKAKSIQKG : 413
gb|KFP53434.1|      : SQTIISYSTQERDNELVMTVG-----TDVGLWIGGHFISFPLYHKAQDWLHYCMAWASQSGMANLWLNGAAGKAKSIQKG : 413
ref|XP_009323546.1| : SQTVLSYSTQERDNELVMTVG-----TDVGLWIGGHFISFPLYHKAQDWLHYCMAWASQSGMANLWLNGAAGKAKSIQKG : 442
gb|KFM01971.1|      : SQTVLSYSTQERDNELVMTVG-----TDVGLWIGGHFISFPLYHKAQDWLHYCMAWASQSGMANLWLNGAAGKAKSIQKG : 413
                        tv sYs    dnEl    g       vglwig  f   p       W  yC  W S  G a lw nG  g        g
```

```
                                *         500         *         520         *         540         *         560
ref|XP_012726090.1| : YIISHCGTFILGKDQDGFLGVSNADGFVGKMTDVNVWDYVITKAEIRDQFLCGKNSTVVGNMLSWGSSKFSMYGGVQLDN : 528
ref|XP_007570993.1| : HMINLAGEFVLGKDQDGVLGISDSDAFVGKMTDVNVWDYVLTTAEIRDQVLCESNSTVVGNVLSWGSSRFKMYGGVQLDN : 534
ref|XP_008411549.1| : HRINLAGAFVLGKDQDGVLGISDSDAFVGKMTDVNVWDYVLTTAEIRDQVLCERNSTVVGNVLSWGSSRFKMYGGVQLDN : 534
ref|XP_004070439.1| : YTIHPGGEFILGKDQDGVLGLSDADAFVGKMTDVNVWNYVLSTAEIRTQMSCGRNTSTVGNVFSWGSTKLSIYGGVQLDS : 531
emb|CAF93821.1|     : YSVSLSGVFILGRDQDGLLGISNSNAFVGKMTDVNVWDYILSTADIRDQMSCERNSTVVGNVLSWGTTRLGIYGGVELDG : 521
ref|XP_011620264.1| : YSVSLSGVFILGRDQDGVLGISNSNAFVGKMTDVNMWDYRLSTADIRDQMSCEGNSTVVGNVISWGTTRLSLYGGVELDG : 535
ref|XP_013875818.1| : YTISPGGVFILGKDQDGLLGISNDDAFVGKLTDVNVWDYVLSAADIRNQMSCKNTSAVVGNVLSWGSTKLSFYGGVQLDN : 535
ref|XP_005478239.2| : YRIRSGGVFILGKDQDGLLGIFNADAFVGKMTDVNVWDYVLSTADIRDQMQCMNNSTVVGNVFSWGNTKLSMYGGVQLDS : 534
ref|XP_006785611.1| : YTIRSGGVFILGKDQDGLLGIFNADAFVGKMTDVNVWDYVLTTADIRDQMQCINNSMVVGNVFSWGNTKLSMYGGVQLDS : 534
ref|XP_005914985.1| : YTIRSGGVFLLGKDQDGLLGIFNADGFVGKMTDVNVWDYVLTTADIRDQMQCMNNSMVVGNVFSWGNTKLSMYGGVQLDS : 534
ref|XP_004554282.1| : YTIRSGGVFLLGKDQDGLLGIFNADGFVGKMTDVNVWDYVLTTADIRDQMQCMNNSMVVGNVFSWGNTKLSMYGGVQLDS : 534
ref|XP_005733115.1| : YTIRSGGVFLLGKDQDGLLGIFNADGFVGKMTDVNVWDYVLTTADIRDQMQCMNNSMVVGNVFSWGNTKLSMYGGVQLDS : 534
ref|XP_010739228.1| : YTISPGGVFILGKDQDGLLGISNANAFVGKMTDVNVWDYVLTTADIRDQMSCERNSTMVGNVFSWGTTQLSLYGGIELDS : 533
ref|XP_008317045.1| : YTINPGGVFILGKDQDGLLGISNTDAFVGKMTDVNVWDYVLTTADIRDQMSCEKNSTVVGNVFSWGSTELSLFDGVQLDS : 534
ref|XP_010793030.1| : YTISPDGVFIVGKDQDGLLGISNADAFVGKMTDINVWDYVLTTADIRDQLSCETNGTVMGNVFSWGSTELSLFGGVQLDS : 536
ref|XP_008294025.1| : YTVSPGGVFILGKDQDGLLGISNADAFVGKMTDVNVWDYVLTTADIRDQMSCESNSTAVGNVFSWGSTKLSLFGGVQLDS : 534
ref|XP_002666525.1| : YIIPAGGRLILGKDQDGFLGISVNDAFVGHMSDVNIWDYVLTEGEIVEQMSCDNG-KVKGNVLSWGVTQLSLYGGVQLQG : 514
ref|XP_009295179.1| : YIIPAGGRLILGKDQDGFLGISVNDAFVGHMSDVNIWDYVLTEGEIVEQMSCDNG-KVKGNVLSWGVTQLSLYGGVQLQG : 522
ref|XP_007246352.1| : YTIRPGGVLILGKDQDGFLGISDSDAFVGQMTEVNVWDYVLSADDIREQMHCEGK-MAKGNVLSWGITRMSLYGGVQLEN : 522
gb|KPP63671.1|      : YTVQPGGILMLGKDEDGFLGISDMDAFVGKMTDVNVWDYVLSPSEIREQMSCGNS-TMKGNVLNWGVTQLSLYGGVRLVT : 481
ref|XP_012681508.1| : YTVASGGMFILGKDQDGFLGISDQDAFVGHMTDVNVWDYVLSAGEIREQMSCENG-TVRGNVLSWGVTQLSLYGGVQLDT : 494
ref|XP_010902628.1| : YTLSPAGVFILGKDQDGFLGISDADAFMGYMTDINVWDYVLTAAEIREQRSCDNKVPLRGNVFSWGVTPLSLYGGVQLET : 516
ref|XP_013989824.1| : YTVSPAGVFILGKDQDGFLGISDTDAFVGQITDVNVWDYVLNSSEIREQMSCEDTPSPRGNVFSWGVTPLSLYGGVQLET : 527
ref|XP_014002952.1| : YTVSPAGVFILGKDQDGFLGISDMDAFVGQMTDVNLWDYVLSSSEIQEQMSCENTTSPRGNVLSWGVTPMSLYGGVQLET : 527
ref|XP_007900354.1| : HVLRKGGTLLLGKDQDGFEGIF-SDVFVGDMTAVNMWDYVIRPAEIRELMECKHR-WKKGNVIAWGKSAVTLFGGVRVED : 501
ref|NP_001085550.1| : YEIQDKGMILLGKNPDDPLGVF-SSGYVGWISQLHLWNYLLEPEHIKSLNQCNML-VARGNVVAWGETKMSISGGVILEP : 521
ref|XP_005986136.1| : YTIKPGGTLVLGNDRNDVMGNY-SHGFTGEISHFNVWNYVLSTSEIRLTQCKT--EMKGNVVGWGTSAMAVFGGVILKI : 483
ref|XP_007432107.1| : YIIQSGGTPILGKRKNAMLDVF-ADAFSGWMSHVNLWSWLLDQNDIQELTLCKHS-QQKGDVFAWGETHMSLFGGVALGP : 515
ref|XP_006035521.2| : YVIQAGGTLVLGKDRDNLLGIF-SNRFIGRMSHVNLWNKVLSHADIRALALCKRN-ELRGDVISWGETPMSVSGGVVLET : 517
ref|XP_014465858.1| : YVTQAGGTLVLGKDRDNLLGIF-SNRFIGRMSHVNLWNKVLSHADIRALALCKHN-ELRGDVIAWGETPMSVSGGVVLET : 517
ref|XP_005293083.1| : YVSQAGGTLVLGKDRDELLGIF-SNGFSGWMSHVSLWSWVISHADIQALALCRRS-ELKGDVIAWGETPMSLSGGVVLES : 517
ref|XP_006127158.1| : YETQAGGTLVLGKDRDELLGIF-SNGFSGWMSHLSLWSWILSHADIRALALCRQG-ELKGDVIAWGETRMSLSGGVVLES : 517
ref|XP_010213863.1| : YETQAGGTLVLGKDRDTLLGTF-SNGFAGWLTHVNMWSRVLGPAEVRALALCKAA-QAKGDVIAWGETALALVGGVVLRP : 514
ref|XP_005057921.1| : YVSQAGGTVLLGKARDALLGTF-SNGFAGWLSRLGLWGRVLGAAEIRALALCRA--QPRGDIIAWGQTPMALLGGVLLQP : 515
ref|XP_002187446.1| : YVSRAGGTLLLGKARDALLGTF-SNGFGGWLSRVNLWSRVLSAAELRALALCRAG-QPTGDVIAWGQTPMALLGGVLLQP : 517
gb|KFO64797.1|      : YVSQAGGTLVLGKDRDTLLGTF-SNGFAGWMTQVNLWSRVLSAAEVRALALCKAG-QLKGDVIAWGQTPMALLGGVLLHN : 492
ref|XP_425745.4|    : YVSQAGGMLVLGKDRDTLLGTF-SNGFVGWMTHVNLWSQVLSPADVRALAQCKAG-NLKGDIIAWGETPMTLFGGVVLKA : 515
ref|XP_010721064.1| : YVSQAGGKLVLGKDRDTLLGTF-SNGFAGWMTHVNLWSQVLSPADIRALAQCKAG-KLKGDVIAWGETPVTLFGGVVLEA : 515
gb|KFW85044.1|      : YVSQAGGMLVLGKDRDTLLGTF-SNGFAGWLAQVNLWSHVLSPAEVRALALCKLG-QPRGDVIAWGQTPMALLGGVVLVA : 491
ref|XP_013049674.1| : YVSQAGGTLVLGKDRDTLLGAF-SNGFAGWMTHVNLWSQVLSPADIRALALCRAG-HPKGDIVAWGETSMTLLGGVVLES : 540
gb|KFQ22427.1|      : YVSQAGGTLVLGKDRDTLLGTF-SNGFAGWLAHVNLWSRVLGPADVRALALCKPG-QLKGDTIAWGETSMTLLGGVVLEP : 491
ref|XP_005143337.1| : YVSQAGGTLVLGKDRDTLLGTF-SNGFAGWMTHVNLWSQVLSPADIRAHALCKPG-HLKGDIIAWGETPMTLLGGVVLEP : 527
gb|KFQ51874.1|      : YVSQAGGTLVLGKDRDTLLGTF-SNGFAGWMTHVNLWSQVLSPLDIRALALCKPG-HMKGDIIAWGETPMTLLGGVVLEP : 491
gb|KFP87788.1|      : YVSQAGGTFVLGKDRDTLLGTF-SNGFAGWMTQVNLWSQVLSPADIRALALCKPG-QLKGDIIAWGETSMALLGGVVLES : 491
gb|KFP08430.1|      : YVSQAGGTLVLGKDRDTLFGTF-SNSFAGWMTQVNLWSQALSPADVRALALCKPG-QLKGDIIAWGETPMTLLGGVVLQS : 491
gb|KFP31739.1|      : YVSQAGGTLVLGKDRDILLGTF-SNGFAGWMTQVNLWSQVLSAADVRALALCKAG-QLKGDIIAWGETPMTLLGGVVLEP : 491
gb|EMC79327.1|      : YVSQAGGTLVLGKDRDTLLGTF-SNGFAGWMTHVNLWSQVLSPADVRTLALCKPG-QLKGDIIAWGEAPMTLFGGVVLES : 515
ref|XP_005237901.1| : YVSQAGGTLVLGKDRDTLLGTF-SNGFAGWMTHVNLWSQVLSPADVRALALCKPG-QTKGDIIAWGETSMTLFGGVVLES : 517
gb|KFV72304.1|      : YVSQAGGTLVLGKDRDILLGTF-SNGFAGWMTHVNLWSQVLSAADVRALALCKPS-QLKGDIIAWGETSMALLGGVVLES : 491
gb|KFU95365.1|      : YVSQAGGTLVLGKDRDTLLGTF-SNGFAGRMTQVNLWSHVLSPEDIRALALCKPG-QLKGDIISWGETSMTLLGGVVLEC : 491
gb|KFU99210.1|      : YVSQAGGTLVLGKDRDTLLGTF-SNGFAGWMTQVNLWSQVLSPAEVRALALCKPG-QLKGDIIAWGETSMTLFGGVVLEP : 491
ref|XP_010076305.1| : YVSQAGGTLVLGKDRDTLLGTF-SNGFAGWMTQVNLWSQVLSPAEVRALALCKPG-QLKGDIIAWGETSMTLFGGVVLEP : 528
gb|KFQ39105.1|      : YVSQAGGTLVLGKDRDTLLGTF-SNGFAGWMTQVNLWSQVLSPADIRALALCKPG-QLKGDIIAWGETSMTLLGGVVLES : 497
gb|KFW07663.1|      : YVSQAGGTLVLGKDRDTLLGTF-SNGFAGWMTHVNLWSQVLSPADVRALALCKPG-QLRGDIIAWGETTMTLLGGVVLEP : 490
gb|KFO73096.1|      : YVSQAGGTLVLGKDRDTLLGTF-SNGFAGWMTHVNLWSQVLSPADVRALAMCKPG-QLRGDVIAWGETSMTLFGGVVLES : 491
gb|KFV05980.1|      : YVSQAGGTLVLGKDRDTLLGTF-SNGFAGWMTHVNLWSQVLSPADVRALALCKPG-QLKGDVVAWGETSMTLLGGVVLES : 491
gb|KFO89464.1|      : YVSQAGGTLVLGKDRDTLLGTF-SNGFAGWMTHVNLWSQVLSPADIRALALCKPG-QLKGDIIAWGETSMTLFGGVVLES : 500
gb|KFP34798.1|      : YVSQAGGTLVLGKERDTLLGTF-SNGFAGWMTHVNLWSQVLSPADIRALALCKPG-QLKGNVIAWGETPMTLLGGVVLES : 491
gb|KFW93611.1|      : YVSQAGGMLVLGKDRDTLLGTF-SNGFAGWMTHVNLWSKVLSPADVRALALCKPG-QQKGDIIAWGETSMTLLGGVVLES : 491
gb|KFP22238.1|      : YVSQAGGTVVLGKNRDTLLGTF-SNGFAGWMTHVNLWSQVLSPADVRALALCKPE-QLKGDIIAWGETSMTLSGGVVLES : 491
gb|KFQ78929.1|      : YVSQAGGTLVLGKDRDTLLGTF-SNGFAGWMTHVNLWSQVLSPADIRALALCKAG-QLKGDIIAWGETSMTLLGGVVLEP : 491
gb|KFP87033.1|      : YVSQSGGTLVLGKDRDTLLGTF-SNGFAGWMTQVNLWSQVLSPADVRALALCKQG-QLKGDIIAWGETSMTLLGGVVLES : 490
gb|KGL96941.1|      : YVSQAGGTLVLGKARDTLLGTF-SNGFAGWMTHVNLWSQVLGPADIRALALCKPG-QLQGDVIAWGETSMTLLGGVVLES : 491
gb|KFZ60550.1|      : YVIQAGGTLVLGKARDTLLGTF-SNGFVGWMTQLNLWSQVLSPADVRALALCKPG-QLKGDIIAWGETSVTLFGGVVLES : 491
gb|KFP94842.1|      : YVSQAGGTLVLGKDRDTLLGTF-SNGFAGWMTHVNLWSQVLSAADVRALALCKPG-QLKGDVIAWGETSMTLLGGVVLES : 491
ref|XP_011583597.1| : YVTQAGGTLVLGKDRDTLLGTF-SNGFAGWMTHVNLWSQVLSPADVRALALCKPG-QLKGDIIAWGETSMTLLGGVVLES : 517
gb|KFZ62512.1|      : YVSQAGGMLVLGKDRDTLLGTF-SNSFAGWMTHVNLWSQVLSPADVRALALCKSG-QLKGDIIAWGETSMTLLGGVVLES : 491
gb|KFQ06064.1|      : YVSQAGGTLVLGKARDTLLGTF-SNGFAGWMTHVNLWSQVLSPADVRALALCKPG-QLKGDIIAWGETSMTLLGGVVLES : 491
ref|XP_009965819.1| : YVSQAGGTLVLGKARDTLLGTF-SNGFAGWMTHVNLWSQVLSPADVRALALCKPG-QLKGDIIAWGETSMTLLGGVVLES : 529
gb|KFV47237.1|      : YVSQAGGTLVLGKDRDTLLGTF-SNGFAGWMTHVNLWSQVLSPADVRALALCKPG-QLKGDIIAWGETSMTLLGGVVLES : 491
gb|KFO11499.1|      : YVSQAGGTLVLGKERDTLLGTF-SNSFAGWMTHVNLWSQVLRPADVRALALCKPG-QLKGDIIAWGETSMTLLGGVVLES : 491
gb|KFQ78854.1|      : YVSQAGGTLVLGKDRDTLLGTF-SNSFAGWMTHVNLWSQVLSPADVRALALCKPG-QLKGDIIAWGETSMTLLGGVVLGS : 491
gb|KFW11285.1|      : YVSQAGGTLVLGKDRDTLLGTF-SNGFAGWMTHVNLWSQVLSPADVRALALCKPG-QLKGDIIAWGETSMTLLGGVVLES : 491
gb|KFQ66673.1|      : YVSQAGGTLVLGKDRDILLGTF-SNGFAGWMTQVNLWSQVLSPADVRALALCKPG-QLKGDIIAWGETSMTLLGGVVLES : 491
gb|KFQ93800.1|      : YVSQAGGTLVLGKARDTLLGTF-SNGFAGWMTHVNLWSQVLSPVDVRALALCKPG-QLKGDIIAWGETSMTLLGGVVLES : 491
gb|KFP53434.1|      : YVSQAGGTLVLGKDRDTLLGTF-SNGFAGWMTHVNLWSQVLSPADVRALALCKPG-QLKGDVIAWGETSMTLLGGVVLES : 491
ref|XP_009323546.1| : YVSQAGGMLVLGKDRDTLLGTF-SNGFAGWMTHVNLWSQVLSPADIRALALCKSG-QLKGDIIAWGETSMTLLGGVVLES : 520
gb|KFM01971.1|      : YVSQAGGMLVLGKDRDTLLGTF-SNGFAGWMTHVNLWSQVLSPADVRALALCKSG-QLKGDIIAWGETSMTLLGGVVLES : 491
                      y    gG   lGkd d  lg      f G mt vn W  vl  a  r    C        G    WG t   l gGv l
```

```
                                *
ref|XP_012726090.1| : QYRCS----------- : 533
ref|XP_007570993.1| : QYRCS----------- : 539
ref|XP_008411549.1| : QYRCS----------- : 539
ref|XP_004070439.1| : QYRCI----------- : 536
emb|CAF93821.1|     : QYRCS----------- : 526
ref|XP_011620264.1| : QYRCS----------- : 540
ref|XP_013875818.1| : VYRCS----------- : 540
ref|XP_005478239.2| : QYRCS----------- : 539
ref|XP_006785611.1| : QYRCS----------- : 539
ref|XP_005914985.1| : QYRCS----------- : 539
ref|XP_004554282.1| : QYRCS----------- : 539
ref|XP_005733115.1| : QYRCS----------- : 539
ref|XP_010739228.1| : QYRC------------ : 537
ref|XP_008317045.1| : QYRCN----------- : 539
ref|XP_010793030.1| : QYRC------------ : 540
ref|XP_008294025.1| : QYRCS----------- : 539
ref|XP_002666525.1| : EQVCHRDNNNNRETEK : 530
ref|XP_009295179.1| : EQVCHRDNNNNRETEK : 538
ref|XP_007246352.1| : EHSCS----------- : 527
gb|KPP63671.1|      : DNRCP----------- : 486
ref|XP_012681508.1| : DHRCP----------- : 499
ref|XP_010902628.1| : DYRCP----------- : 521
ref|XP_013989824.1| : DYRCH----------- : 532
ref|XP_014002952.1| : DYRCH----------- : 532
ref|XP_007900354.1| : DNSCLG---------- : 507
ref|NP_001085550.1| : DNSC------------ : 525
ref|XP_005986136.1| : DSSCK----------- : 488
ref|XP_007432107.1| : DTSCR----------- : 520
ref|XP_006035521.2| : DSSCH----------- : 522
ref|XP_014465858.1| : DSSCH----------- : 522
ref|XP_005293083.1| : DPSCQ----------- : 522
ref|XP_006127158.1| : DPSCR----------- : 522
ref|XP_010213863.1| : DTSCE----------- : 519
ref|XP_005057921.1| : DTSCH----------- : 520
ref|XP_002187446.1| : DTSCQ----------- : 522
gb|KFO64797.1|      : DTSC------------ : 496
ref|XP_425745.4|    : DTSCQ----------- : 520
ref|XP_010721064.1| : DTSCQ----------- : 520
gb|KFW85044.1|      : DTSC------------ : 495
ref|XP_013049674.1| : DTSCQ----------- : 545
gb|KFQ22427.1|      : DTSC------------ : 495
ref|XP_005143337.1| : DTSCQ----------- : 532
gb|KFQ51874.1|      : DTSC------------ : 495
gb|KFP87788.1|      : DTSC------------ : 495
gb|KFP08430.1|      : DTSC------------ : 495
gb|KFP31739.1|      : DTSC------------ : 495
gb|EMC79327.1|      : DTSCQ----------- : 520
ref|XP_005237901.1| : DTSCQ----------- : 522
gb|KFV72304.1|      : DTSC------------ : 495
gb|KFU95365.1|      : DTSC------------ : 495
gb|KFU99210.1|      : DTSC------------ : 495
ref|XP_010076305.1| : DTSCQ----------- : 533
gb|KFQ39105.1|      : DTSC------------ : 501
gb|KFW07663.1|      : DTSC------------ : 494
gb|KFO73096.1|      : DTSC------------ : 495
gb|KFV05980.1|      : DTSC------------ : 495
gb|KFO89464.1|      : DTSC------------ : 504
gb|KFP34798.1|      : DTSC------------ : 495
gb|KFW93611.1|      : DTSC------------ : 495
gb|KFP22238.1|      : DTSC------------ : 495
gb|KFQ78929.1|      : DTSC------------ : 495
gb|KFP87033.1|      : DTSC------------ : 494
gb|KGL96941.1|      : DTSC------------ : 495
gb|KFZ60550.1|      : DTSC------------ : 495
gb|KFP94842.1|      : DTSC------------ : 495
ref|XP_011583597.1| : DSSCQ----------- : 522
gb|KFZ62512.1|      : DTS------------- : 494
gb|KFQ06064.1|      : DTSC------------ : 495
ref|XP_009965819.1| : DTSCQ----------- : 534
gb|KFV47237.1|      : DTSC------------ : 495
gb|KFO11499.1|      : DTSC------------ : 495
gb|KFQ78854.1|      : DTSC------------ : 495
gb|KFW11285.1|      : DTSC------------ : 495
gb|KFQ66673.1|      : DTSC------------ : 495
gb|KFQ93800.1|      : DTSC------------ : 495
gb|KFP53434.1|      : DTSC------------ : 495
ref|XP_009323546.1| : DTSCQ----------- : 525
gb|KFM01971.1|      : DTSC------------ : 495
                         c
```
