# Supplementary figures and images for: Identification and characterization of a novel zebrafish (Danio rerio) pentraxin–carbonic anhydrase
Source: PeerJ. 2017 Dec 7;5:e4128. doi: 10.7717/peerj.4128 (PMC5723433; doi:10.7717/peerj.4128)

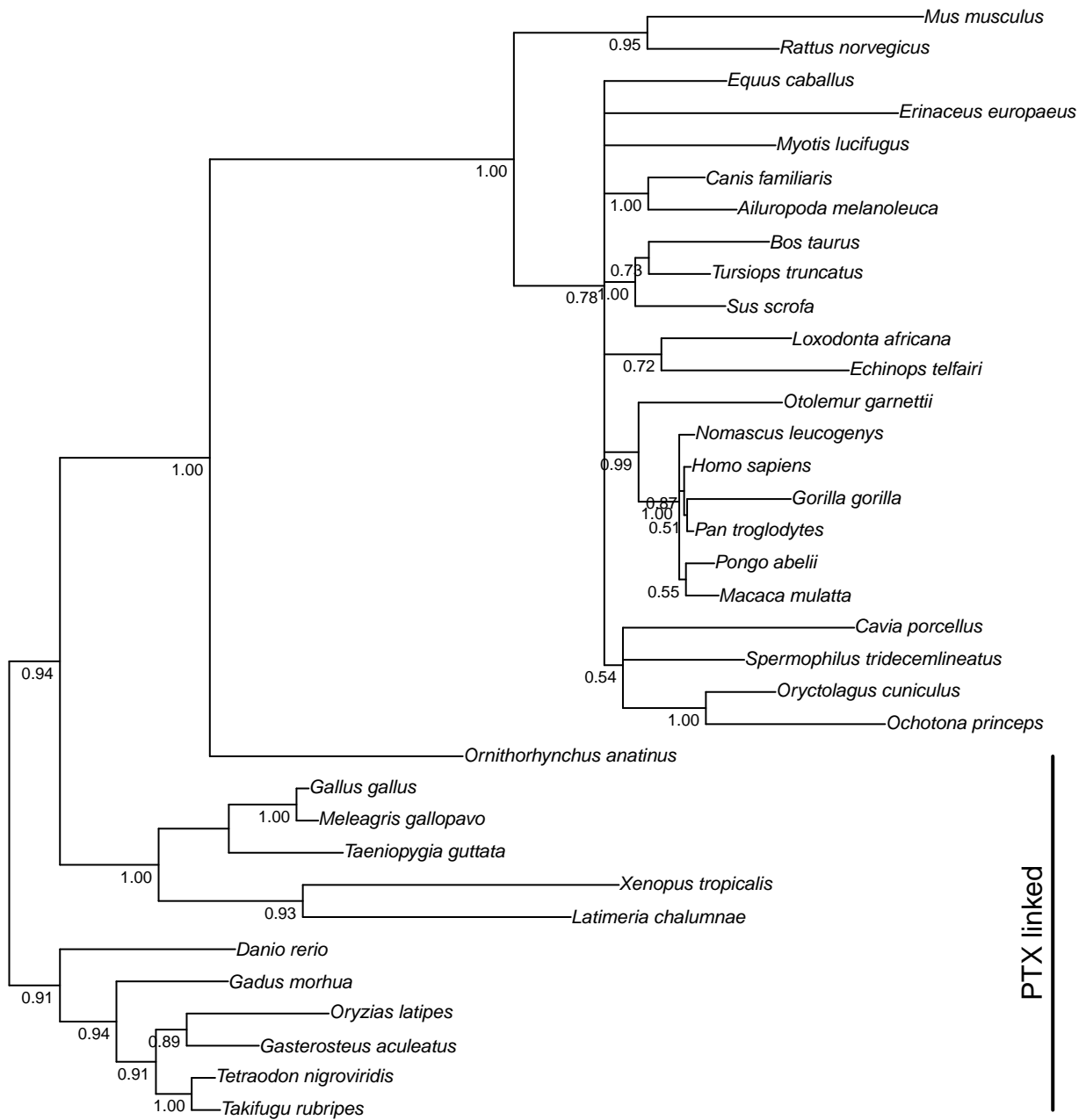

PTX linked

Supplement: Supplemental Information 8 — Analysis of protein alignment guided DNA alignments as detailed in Materials and methods. Sidebar indicates species in which the presence of a PTX domain in CA VI is observed (non-mammals) or assumed (O. anatus). [file peerj-05-4128-s008.pdf]

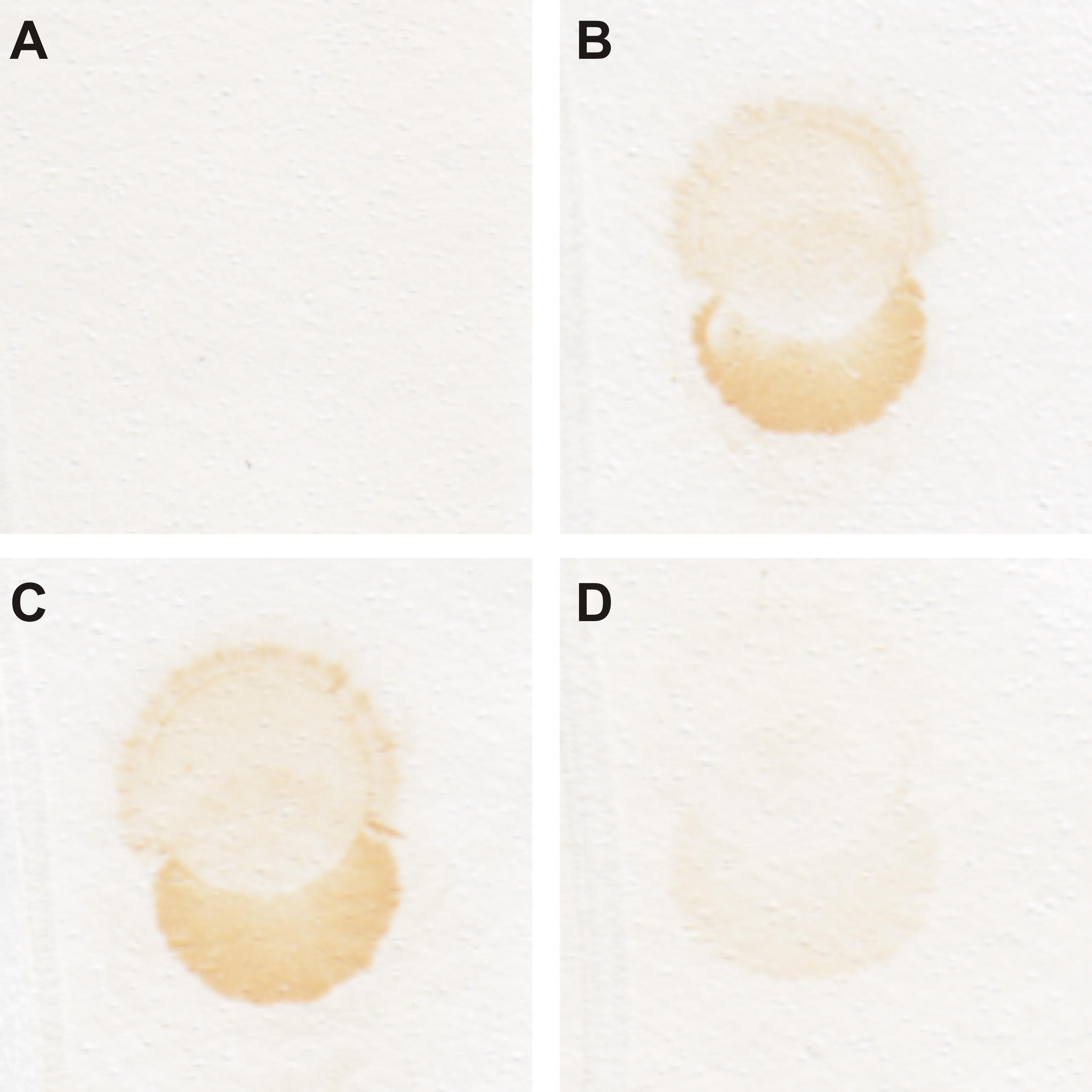

Supplement: Supplemental Information 9 — 500 ng of purified recombinant zebrafish CA VI-PTX antigen was attached as spots on nitrocellulose, incubated with antisera from various stages of the immunization process, and stained with a peroxidase-coupled secondary antibody. A) Pre-immune serum, no recognition of the CA VI-PTX protein; B) Bleed 1 (day 41); C) Bleed 2 (day 62); and D) Bleed 3 (day 83). In B and C the antiserum strongly recognizes the recombinant protein, whereas the signal is clearly diminished when bleed 3 (D) was tested. [file peerj-05-4128-s009.png]

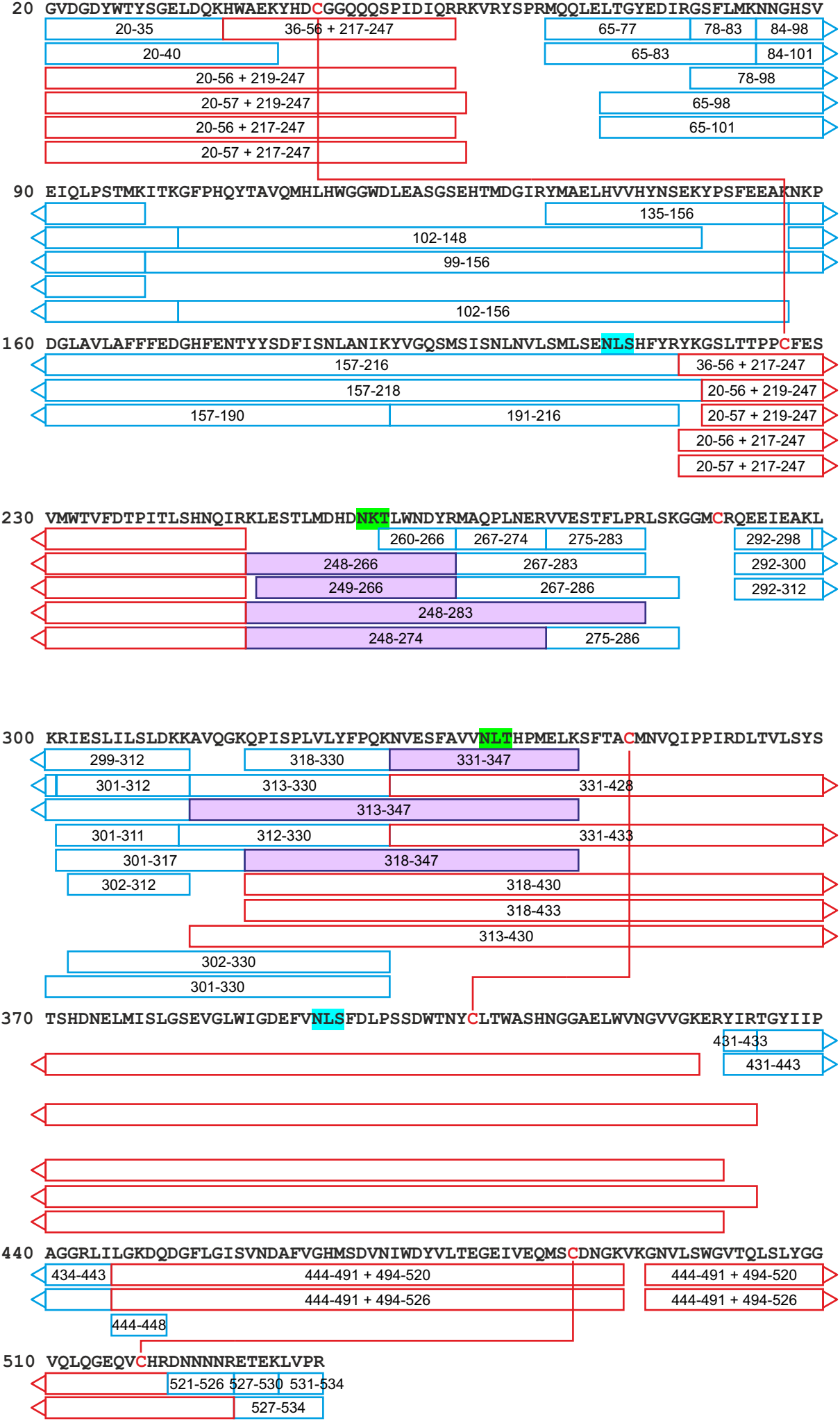

Supplement: Supplemental Information 12 [file peerj-05-4128-s012.pdf]

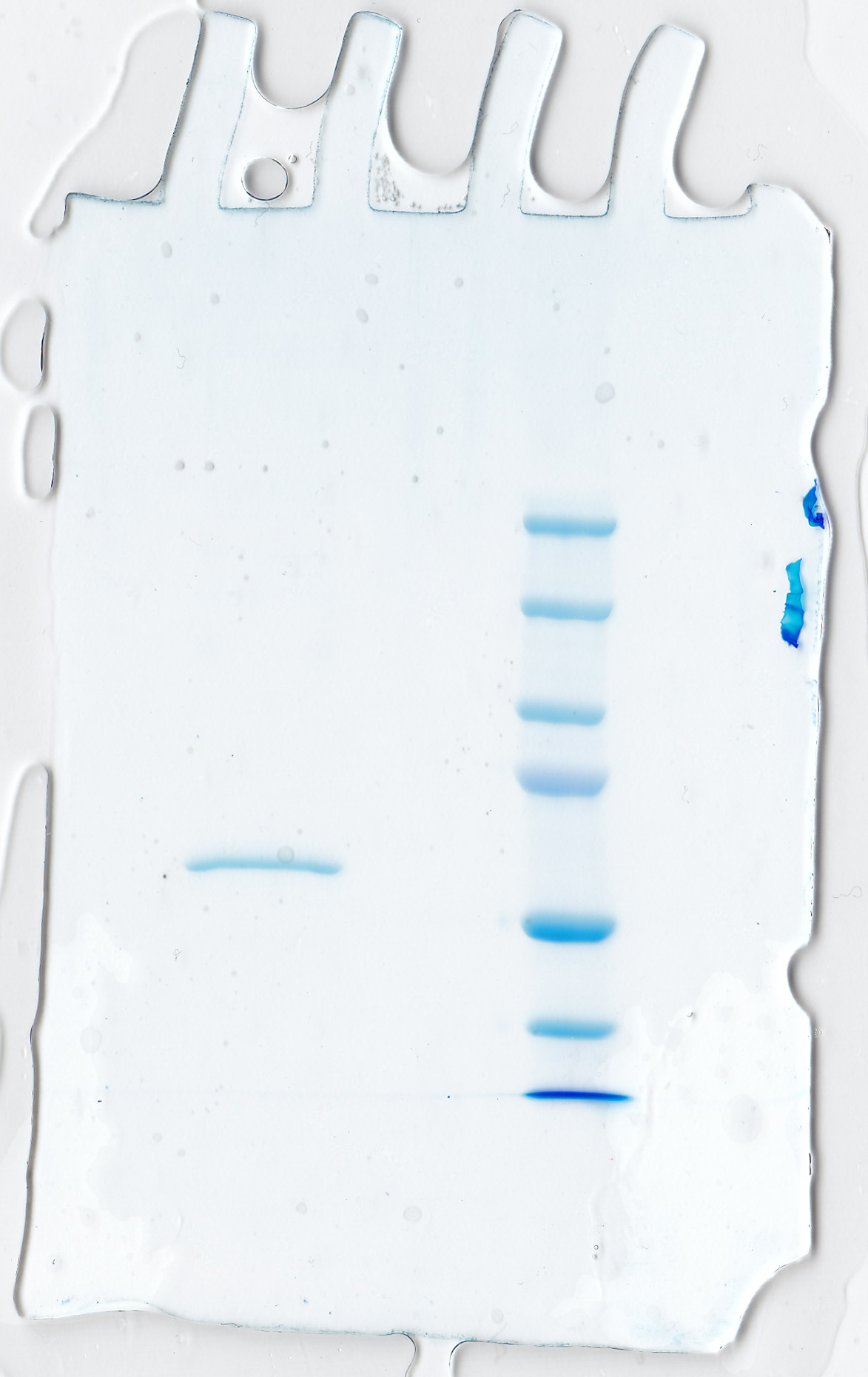

Supplement: Supplemental Information 14 [file peerj-05-4128-s014.png]
