# Supplementary material for: Identification and characterization of a novel zebrafish (Danio rerio) pentraxin–carbonic anhydrase
Source: PeerJ. 2017 Dec 7;5:e4128. doi: 10.7717/peerj.4128 (PMC5723433; doi:10.7717/peerj.4128)
Supplement: Supplemental Information 10 — ca6zf = sequence of the PCR amplification product of this study [submitted to ENA database (http://www.ebi.ac.uk/ena) as LT724251], CA6 = coding sequence of transcript ENSDART00000132733 from Ensembl. Translated sequences are shown aligned in Fig. S5. [file peerj-05-4128-s010.pdf]

```
                 *        20          *        40          *        60          *        80
ca6zf : ATGGAGCAGCTGACTCTAGTCCTGCTNTTCNNCACTTCGCTGAATTTCGCATCGGCTGGAGTAGATGGAGATTATTGGAC :   80
CA6   : ATGGAGCAGCTGACTCTAGTCCTGCTATTCGTCACTTCGCTGAATTTCGCATCGGCTGGAGTAGATGGAGATTATTGGAC :   80
        ATGGAGCAGCTGACTCTAGTCCTGCT TTC  CACTTCGCTGAATTTCGCATCGGCTGGAGTAGATGGAGATTATTGGAC

                 *       100          *       120          *       140          *       160
ca6zf : ATATTCAGGAGAGCTGGATCAGAAGCACTGGGCAGAAAAATATCATGACTGCGGTGGACAACAACAGTCTCCCATTGACA :  160
CA6   : ATATTCAGGAGAGCTGGATCAGAAGCACTGGGCAGAAAAATATCATGACTGCGGTGGCCAACAACAGTCTCCCATTGACA :  160
        ATATTCAGGAGAGCTGGATCAGAAGCACTGGGCAGAAAAATATCATGACTGCGGTGG CAACAACAGTCTCCCATTGACA

                 *       180          *       200          *       220          *       240
ca6zf : TACAGCGGCGAAAGGTGCGATATAGTCCACGAATGCAACAGCTCGAGCTGACAGGTTATGAAGACATCCGCGGTTCCTTT :  240
CA6   : TACAGCGGCGAAAGGTGCGATATAGTCCACGAATGCAACAGCTCGAGCTGACAGGTTATGAAGACATCCGCGGTTCCTTT :  240
        TACAGCGGCGAAAGGTGCGATATAGTCCACGAATGCAACAGCTCGAGCTGACAGGTTATGAAGACATCCGCGGTTCCTTT

                 *       260          *       280          *       300          *       320
ca6zf : CTCATGAAGAACAATGGCCATTCTGTGGAAATTCAGCTGCCAAGCACCATGAAGATTACCAAGGGGTTTCCACACCAATA :  320
CA6   : CTCATGAAGAACAATGGCCATTCTGTGGAAATTCAGCTGCCAAGCACCATGAAGATTACCAAGGGGTTTCCACACCAATA :  320
        CTCATGAAGAACAATGGCCATTCTGTGGAAATTCAGCTGCCAAGCACCATGAAGATTACCAAGGGGTTTCCACACCAATA

                 *       340          *       360          *       380          *       400
ca6zf : CACCGCCGTCCAAATGCATCTGCACTGGGGAGGCTGGGACTTGGAGGCCAGTGGATCAGAGCACACAATGGACGGCATTC :  400
CA6   : CACCGCCGTCCAAATGCATCTGCACTGGGGAGGCTGGGACTTGGAGGCCAGTGGATCAGAGCACACAATGGACGGCATTC :  400
        CACCGCCGTCCAAATGCATCTGCACTGGGGAGGCTGGGACTTGGAGGCCAGTGGATCAGAGCACACAATGGACGGCATTC

                 *       420          *       440          *       460          *       480
ca6zf : GCTACATGGCAGAACTTCACGTTGTCCACTACAACTCTGAAAAGTACCCCAGCTTTGAAGAGGCCAAAAATAAACCTGAT :  480
CA6   : GCTACATGGCAGAACTTCACGTTGTCCACTACAACTCTGAAAAGTACCCCAGCTTTGAAGAAGCCAAAAATAAACCTGAT :  480
        GCTACATGGCAGAACTTCACGTTGTCCACTACAACTCTGAAAAGTACCCCAGCTTTGAAGA GCCAAAAATAAACCTGAT

                 *       500          *       520          *       540          *       560
ca6zf : GGTCTTGCAGTTTTAGCCTTCTTTTTTGAGGATGGACATTTTGAGAACACGTACTACAGTGATTTTATTTCAAACCTGGC :  560
CA6   : GGTCTTGCAGTTTTAGCCTTCTTTTTTGAGGATGGGCATTTTGAGAACACGTACTACAGTGATTTTATTTCAAACCTGGC :  560
        GGTCTTGCAGTTTTAGCCTTCTTTTTTGAGGATGG CATTTTGAGAACACGTACTACAGTGATTTTATTTCAAACCTGGC

                 *       580          *       600          *       620          *       640
ca6zf : AAACATCAAATACGTAGGTCAGTCCATGAGCATCTCCAACCTGAATGTTCTCTCCATGCTGTCAGAGAACCTTAGTCACT :  640
CA6   : AAACATCAAATACGTAGGTCAGTCCATGAGCATCTCCAACCTGAATGTTCTCTCCATGCTGTCAGAGAACCTTAGTCACT :  640
        AAACATCAAATACGTAGGTCAGTCCATGAGCATCTCCAACCTGAATGTTCTCTCCATGCTGTCAGAGAACCTTAGTCACT

                 *       660          *       680          *       700          *       720
ca6zf : TCTACAGATATAAAGGCTCTCTGACCACACCGCCCTGTTTTGAGAGCGTCATGTGGACAGTGTTTGATACGCCCATCACC :  720
CA6   : TCTACAGATATAAAGGCTCTCTGACCACACCGCCCTGTTTTGAGAGCGTCATGTGGACAGTGTTCGATACGCCGATCACC :  720
        TCTACAGATATAAAGGCTCTCTGACCACACCGCCCTGTTTTGAGAGCGTCATGTGGACAGTGTT GATACGCC ATCACC

                 *       740          *       760          *       780          *       800
ca6zf : CTCTCGCACAACCAGATCAGGAAATTGGAGAGCACATTAATGGACCATGACAATAAGACCCTGTGGAACGACTACCGCAT :  800
CA6   : CTCTCGCACAACCAGATCAGGAAATTGGAGAGCACATTAATGGACCATGACAATAAGACCCTGTGGAACGACTACCGCAT :  800
        CTCTCGCACAACCAGATCAGGAAATTGGAGAGCACATTAATGGACCATGACAATAAGACCCTGTGGAACGACTACCGCAT

                 *       820          *       840          *       860          *       880
ca6zf : GGCCCAACCTCTGAATGAAAGAGTGGTGGAGTCTACTTTCCTCCCACGTCTCAGCAAAGGAGGAATGTGCCGTCAAGAGG :  880
CA6   : GGCCCAACCTCTGAATGAAAGAGTGGTGGAGTCTACTTTCCTCCCACGTCTCAGCAAAGGAGGAATGTGCCGTCAAGAGG :  880
        GGCCCAACCTCTGAATGAAAGAGTGGTGGAGTCTACTTTCCTCCCACGTCTCAGCAAAGGAGGAATGTGCCGTCAAGAGG

                 *       900          *       920          *       940          *       960
ca6zf : AGATTGAAGCTAAGCTTAAAAGGATCGAGAGCCTTATTTTGTCACTGGACAAAAAAGCGGTTCAAGGCAAGCAACCTATA :  960
CA6   : AGATTGAAGCTAAGCTTAAAAGGATCGAGAGCCTTATTTTGTCACTGGACAAAAAAGCGGTTCAAGGCAAGCAACCTATA :  960
        AGATTGAAGCTAAGCTTAAAAGGATCGAGAGCCTTATTTTGTCACTGGACAAAAAAGCGGTTCAAGGCAAGCAACCTATA

                 *       980          *      1000          *      1020          *      1040
ca6zf : TCTCCCCTGGTCCTCTACTTCCCGCAGAAAAAATGTGGAAAGCTTCGCCGTGGTGAACTTAACACATCCCATGGAGCTCAA : 1040
CA6   : TCTCCCCTGGTCCTCTACTTCCCGCAGAAAAAATGTGGAAAGCTTCGCCGTGGTGAACTTAACACATCCCATGGAGCTCAA : 1040
        TCTCCCCTGGTCCTCTACTTCCCGCAGAAAAAATGTGGAAAGCTTCGCCGTGGTGAACTTAACACATCCCATGGAGCTCAA

                 *      1060          *      1080          *      1100          *      1120
ca6zf : ATCCTTCACAGCCTGCATGAATGTCCAAATTCCCCCAATTCGAGATCTGACTGTGCTTTCATACTCCACGTCCCACGACA : 1120
CA6   : ATCCTTCACAGCCTGCATGAATGTCCAAATTCCCCCAATTCGAGATCTGACTGTGCTTTCATACTCCACGTCCCACGACA : 1120
        ATCCTTCACAGCCTGCATGAATGTCCAAATTCCCCCAATTCGAGATCTGACTGTGCTTTCATACTCCACGTCCCACGACA
```

```
                   *      1140          *      1160          *      1180          *      1200
ca6zf : ATGAGCTCATGATCTCCTTAGGCTCTGAAGTGGGTCTCTGGATCGGAGATGAGTTTGTAAATCTGAGTTTCGATCTTCCA : 1200
CA6   : ATGAGCTCATGATCTCCTTAGGCTCTGAAGTGGGTCTCTGGATCGGAGATGAGTTTGTAAATCTGAGTTTCGATCTTCCA : 1200
        ATGAGCTCATGATCTCCTTAGGCTCTGAAGTGGGTCTCTGGATCGGAGATGAGTTTGTAAATCTGAGTTTCGATCTTCCA

                   *      1220          *      1240          *      1260          *      1280
ca6zf : TCAAGTGACTGGACAAACTACTGCCTTACCTGGGCATCACACAACGGAGGGGCTGAGCTGTGGGTGAACGGGGTAGTCGG : 1280
CA6   : TCAAGTGACTGGACAAACTACTGCCTTACCTGGGCATCACACAACGGAGGGGCTGAGCTGTGGGTGAACGGGGTAGTCGG : 1280
        TCAAGTGACTGGACAAACTACTGCCTTACCTGGGCATCACACAACGGAGGGGCTGAGCTGTGGGTGAACGGGGTAGTCGG

                   *      1300          *      1320          *      1340          *      1360
ca6zf : AAAGGAACGCTACATTAGGACAGGGTACATCATTCCTGCTGGAGGAAGACTCATTTTGGGGAAGGACCAGGACGGGTTTT : 1360
CA6   : AAAGGAACGCTACATTAGGACAGGGTACATCATTCCTGCTGGAGGAAGACTCATTTTGGGGAAGGACCAGGACGGGTTTT : 1360
        AAAGGAACGCTACATTAGGACAGGGTACATCATTCCTGCTGGAGGAAGACTCATTTTGGGGAAGGACCAGGACGGGTTTT

                   *      1380          *      1400          *      1420          *      1440
ca6zf : TAGGGATTTCAGTTAACGATGCTTTTGTCGGTCATATGAGTGATGTAAACATTTGGGATTATGTGTTAACTGAGGGAGAG : 1440
CA6   : TAGGGATTTCAGTTAACGATGCTTTTGTCGGTCATATGAGTGATGTAAACATTTGGGATTATGTGTTAACTGAGGGAGAG : 1440
        TAGGGATTTCAGTTAACGATGCTTTTGTCGGTCATATGAGTGATGTAAACATTTGGGATTATGTGTTAACTGAGGGAGAG

                   *      1460          *      1480          *      1500          *      1520
ca6zf : ATTGTTGAGCAGATGTCGTGTGACAATGGGAAAGTGAAGGGGAACGTCCTGAGCTGGGGAGTCACTCAGCTCAGTCTGTA : 1520
CA6   : ATTGTTGAGCAGATGTCGTGTGACAATGGGAAAGTGAAGGGGAACGTCCTGAGCTGGGGAGTCACTCAGCTCAGTCTGTA : 1520
        ATTGTTGAGCAGATGTCGTGTGACAATGGGAAAGTGAAGGGGAACGTCCTGAGCTGGGGAGTCACTCAGCTCAGTCTGTA

                   *      1540          *      1560          *      1580          *
ca6zf : TGGAGGAGTTCAGCTGCAGGGCGAACAGGTTTGCCATCGGGATAATAATAATAATAGAGAAACAGAGAAA : 1590
CA6   : TGGAGGAGTTCAGCTGCAGGGCGAACAGGTTTGCCATCGGGATAATAATAATAATAGAGAAACAGAGAAA : 1590
        TGGAGGAGTTCAGCTGCAGGGCGAACAGGTTTGCCATCGGGATAATAATAATAATAGAGAAACAGAGAAA
```
