# Supplementary material for: Identification and characterization of a novel zebrafish (Danio rerio) pentraxin–carbonic anhydrase
Source: PeerJ. 2017 Dec 7;5:e4128. doi: 10.7717/peerj.4128 (PMC5723433; doi:10.7717/peerj.4128)
Supplement: Supplemental Information 11 — Comparison to a reference sequence. ca6zf = protein translation of the PCR amplification product of this study (UniProt A0A1R4AHH7), CA6 = reference protein sequence (UniProt E9QB97). Underlined N-terminal sequences: Signal peptides as reported in UniProt and as confirmed by mass spectrometry in this study. [file peerj-05-4128-s011.pdf]

```
ca6zf    MEQLTLVLLFXTSLNFASAGVDGDYWTYSGELDQKHWAEKYHDCGGQQQSPIDIQRRKVR
CA6      MEQLTLVLLFVTSLNFASAGVDGDYWTYSGELDQKHWAEKYHDCGGQQQSPIDIQRRKVR
         ********** *************************************************

ca6zf    YSPRMQQLELTGYEDIRGSFLMKNNGHSVEIQLPSTMKITKGFPHQYTAVQMHLHWGGWD
CA6      YSPRMQQLELTGYEDIRGSFLMKNNGHSVEIQLPSTMKITKGFPHQYTAVQMHLHWGGWD
         ************************************************************

ca6zf    LEASGSEHTMDGIRYMAELHVVHYNSEKYPSFEEAKNKPDGLAVLAFFFEDGHFENTYYS
CA6      LEASGSEHTMDGIRYMAELHVVHYNSEKYPSFEEAKNKPDGLAVLAFFFEDGHFENTYYS
         ************************************************************

ca6zf    DFISNLANIKYVGQSMSISNLNVLSMLSENLSHFYRYKGSLTTPPCFESVMWTVFDTPIT
CA6      DFISNLANIKYVGQSMSISNLNVLSMLSENLSHFYRYKGSLTTPPCFESVMWTVFDTPIT
         ************************************************************

ca6zf    LSHNQIRKLESTLMDHDNKTLWNDYRMAQPLNERVVESTFLPRLSKGGMCRQEEIEAKLK
CA6      LSHNQIRKLESTLMDHDNKTLWNDYRMAQPLNERVVESTFLPRLSKGGMCRQEEIEAKLK
         ************************************************************

ca6zf    RIESLILSLDKKAVQGKQPISPLVLYFPQKNVESFAVVNLTHPMELKSFTACMNVQIPPI
CA6      RIESLILSLDKKAVQGKQPISPLVLYFPQKNVESFAVVNLTHPMELKSFTACMNVQIPPI
         ************************************************************

ca6zf    RDLTVLSYSTSHDNELMISLGSEVGLWIGDEFVNLSFDLPSSDWTNYCLTWASHNGGAEL
CA6      RDLTVLSYSTSHDNELMISLGSEVGLWIGDEFVNLSFDLPSSDWTNYCLTWASHNGGAEL
         ************************************************************

ca6zf    WVNGVVGKERYIRTGYIIPAGGRLILGKDQDGFLGISVNDAFVGHMSDVNIWDYVLTEGE
CA6      WVNGVVGKERYIRTGYIIPAGGRLILGKDQDGFLGISVNDAFVGHMSDVNIWDYVLTEGE
         ************************************************************

ca6zf    IVEQMSCDNGKVKGNVLSWGVTQLSLYGGVQLQGEQVCHRDNNNNRETEK
CA6      IVEQMSCDNGKVKGNVLSWGVTQLSLYGGVQLQGEQVCHRDNNNNRETEK
         **************************************************
```
