# Supplementary material for: Identification and characterization of a novel zebrafish (Danio rerio) pentraxin–carbonic anhydrase
Source: PeerJ. 2017 Dec 7;5:e4128. doi: 10.7717/peerj.4128 (PMC5723433; doi:10.7717/peerj.4128)
Supplement: Supplementary file 11 [file peerj-05-4128-s013.docx]

List of peptides from zebrafish CA VI-PTX, observed by mass spectrometry

Experimental	Theoretical	Error (ppm)	Residues	Sequence
450,2588	450,2591	-0,69	431-433	YIR
483,3168	483,3169	-0,21	531-534	LVPR
505,2382	505,2384	-0,4	527-530	ETEK
542,3791	542,3792	-0,26	444-448	LILGK
681,3524	681,352	0,59	78-83	GSFLMK
745,3107	745,3103	0,52	521-526	DNNNNR
845,4128	845,4131	-0,41	292-298	QEEIEAK
957,4707	957,4702	0,54	267-274	MAQPLNER
966,4566	966,4559	0,74	260-266	TLWNDYR
970,5449	970,5447	0,23	527-534	ETEKLVPR
1003,5454	1003,5451	0,27	434-443	TGYIIPAGGR
1046,5763	1046,576	0,26	275-283	VVESTFLPR
1086,5921	1086,5921	-0,01	292-300	QEEIEAKLK
1257,7552	1257,7544	0,63	302-312	IESLILSLDKK
1285,7605	1285,7606	-0,1	301-311	RIESLILSLDK
1374,7877	1374,7871	0,41	275-286	VVESTFLPRLSK
1413,8548	1413,8555	-0,48	301-312	RIESLILSLDKK
1435,7945	1435,7936	0,65	431-443	YIRTGYIIPAGGR
1528,8664	1528,8654	0,63	318-330	QPISPLVLYFPQK
1594,7666	1594,7661	0,31	65-77	MQQLELTGYEDIR
1653,8157	1653,8145	0,74	84-98	NNGHSVEIQLPSTMK
1655,035	1655,0345	0,33	299-312	LKRIESLILSLDKK
1831,7915	1831,7901	0,79	20-35	GVDGDYWTYSGELDQK
1897,1367	1897,1361	0,34	301-317	RIESLILSLDKKAVQGK
1986,0368	1986,0357	0,57	267-283	MAQPLNERVVESTFLPR
1996,0423	1996,0412	0,56	84-101	NNGHSVEIQLPSTMKITK
2012,1471	2012,1459	0,6	313-330	AVQGKQPISPLVLYFPQK
2140,2415	2140,2409	0,29	312-330	KAVQGKQPISPLVLYFPQK
2258,1083	2258,1075	0,33	65-83	MQQLELTGYEDIRGSFLMK
2314,2477	2314,2467	0,43	267-286	MAQPLNERVVESTFLPRLSK
2317,1571	2317,1559	0,5	78-98	GSFLMKNNGHSVEIQLPSTMK
2482,438	2482,437	0,42	292-312	QEEIEAKLKRIESLILSLDKK
2483,1048	2483,103	0,72	20-40	GVDGDYWTYSGELDQKHWAEK
2670,2444	2670,2424	0,74	135-156	YMAELHVVHYNSEKYPSFEEAK
2819,3059	2819,3046	0,45	331-347	NVESFAVVNLTHPMELK + 2xGlcNAc+3xMan
2988,448	2988,4474	0,19	191-216	YVGQSMSISNLNVLSMLSENLSHFYR
3251,8921	3251,8897	0,73	302-330	IESLILSLDKKAVQGKQPISPLVLYFPQK
3288,4121	3288,4127	-0,19	249-266	LESTLMDHDNKTLWNDYR + 2xGlcNAc+1xFuc+3xMan
3407,9923	3407,9908	0,43	301-330	RIESLILSLDKKAVQGKQPISPLVLYFPQK
3416,5084	3416,5076	0,23	248-266	KLESTLMDHDNKTLWNDYR + 2xGlcNAc+1xFuc+3xMan
3893,9112	3893,9114	-0,04	65-98	MQQLELTGYEDIRGSFLMKNNGHSVEIQLPSTMK
3895,87	3895,8734	-0,87	157-190	NKPDGLAVLAFFFEDGHFENTYYSDFISNLANIK
4236,1396	4236,1381	0,36	65-101	MQQLELTGYEDIRGSFLMKNNGHSVEIQLPSTMKITK
4330,1608	4330,1594	0,32	318-347	QPISPLVLYFPQKNVESFAVVNLTHPMELK + 2xGlcNAc+3xMan
4355,9705	4355,9673	0,74	248-274	KLESTLMDHDNKTLWNDYRMAQPLNER + 2xGlcNAc+1xFuc+3xMan
4813,4427	4813,4399	0,58	313-347	AVQGKQPISPLVLYFPQKNVESFAVVNLTHPMELK + 2xGlcNAc+3xMan
5384,5407	5384,5328	1,46	248-283	KLESTLMDHDNKTLWNDYRMAQPLNERVVESTFLPR + 2xGlcNAc+1xFuc+3xMan
5450,4852	5450,4873	-0,39	102-148	GFPHQYTAVQMHLHWGGWDLEASGSEHTMDGIRYMAELHVVHYNSEK
6060,8846	6060,8775	1,16	36-56 + 217-247	HWAEKYHDCGGQQQSPIDIQR + YKGSLTTPPCFESVMWTVFDTPITLSHNQIR
6401,917	6401,9211	-0,64	102-156	GFPHQYTAVQMHLHWGGWDLEASGSEHTMDGIRYMAELHVVHYNSEKYPSFEEAK
6744,1475	6744,1478	-0,05	99-156	ITKGFPHQYTAVQMHLHWGGWDLEASGSEHTMDGIRYMAELHVVHYNSEKYPSFEEAK
6866,3024	6866,3102	-1,13	157-216	NKPDGLAVLAFFFEDGHFENTYYSDFISNLANIKYVGQSMSISNLNVLSMLSENLSHFYR
7157,4688	7157,4685	0,04	157-218	NKPDGLAVLAFFFEDGHFENTYYSDFISNLANIKYVGQSMSISNLNVLSMLSENLSHFYRYK
7583,503	7583,4987	0,56	20-56 + 219-247	GVDGDYWTYSGELDQKHWAEKYHDCGGQQQSPIDIQR + GSLTTPPCFESVMWTVFDTPITLSHNQIR
7739,608	7739,5998	1,06	20-57 + 219-247	GVDGDYWTYSGELDQKHWAEKYHDCGGQQQSPIDIQRRK + GSLTTPPCFESVMWTVFDTPITLSHNQIR
7874,6599	7874,657	0,37	20-56 + 217-247	GVDGDYWTYSGELDQKHWAEKYHDCGGQQQSPIDIQR + YKGSLTTPPCFESVMWTVFDTPITLSHNQIR
8030,7518	8030,7581	-0,78	20-57 + 217-247	GVDGDYWTYSGELDQKHWAEKYHDCGGQQQSPIDIQRR + YKGSLTTPPCFESVMWTVFDTPITLSHNQIR
8195,9294	8195,9442	-1,81	444-491 + 494-520	LILGKDQDGFLGISVNDAFVGHMSDVNIWDYVLTEGEIVEQMSCDNGK + GNVLSWGVTQLSLYGGVQLQGEQVCHR
8923,2284	8923,244	-1,74	444-491 + 494-526	LILGKDQDGFLGISVNDAFVGHMSDVNIWDYVLTEGEIVEQMSCDNGK + GNVLSWGVTQLSLYGGVQLQGEQVCHRDNNNNR
11701,5231	11701,5228	0,02	331-428	NVESFAVVNLTHPMELKSFTACMNVQIPPIRDLTVLSYSTSHDNELMISLGSEVGLWIGDEFVNLSFDLPSSDWTNYCLTWASHNGGAELWVNGVVGK + 2xGlcNAc+3xMan
12418,8998	12418,9151	-1,23	331-433	NVESFAVVNLTHPMELKSFTACMNVQIPPIRDLTVLSYSTSHDNELMISLGSEVGLWIGDEFVNLSFDLPSSDWTNYCLTWASHNGGAELWVNGVVGKERYIR + 2xGlcNAc+3xMan
13497,5457	13497,5213	1,81	318-430	QPISPLVLYFPQKNVESFAVVNLTHPMELKSFTACMNVQIPPIRDLTVLSYSTSHDNELMISLGSEVGLWIGDEFVNLSFDLPSSDWTNYCLTWASHNGGAELWVNGVVGKER + 2xGlcNAc+3xMan
13929,7906	13929,7698	1,49	318-433	QPISPLVLYFPQKNVESFAVVNLTHPMELKSFTACMNVQIPPIRDLTVLSYSTSHDNELMISLGSEVGLWIGDEFVNLSFDLPSSDWTNYCLTWASHNGGAELWVNGVVGKERYIR + 2xGlcNAc+3xMan
13980,8166	13980,8019	1,05	313-430	AVQGKQPISPLVLYFPQKNVESFAVVNLTHPMELKSFTACMNVQIPPIRDLTVLSYSTSHDNELMISLGSEVGLWIGDEFVNLSFDLPSSDWTNYCLTWASHNGGAELWVNGVVGKER + 2xGlcNAc+3xMan
